# CCL18: a potential immunosuppressive biomarker for prognosis in ABC diffuse large B-cell lymphoma

**DOI:** 10.3389/fimmu.2025.1693730

**Published:** 2025-12-15

**Authors:** Marta Rodríguez, Francisco Rojas-Vega, Jesus Frutos Díaz-Alejo, Ignacio Mahillo-Fernández, Cristina Serrano, Alberto López, Teresa Morales-Ruiz, Teresa Roldan-Arjona, Joaquín Sánchez-García, Ana Río-Machín, Daniel Morillo, M. Angeles Pérez, Raúl Córdoba, Pilar Llamas-Sillero, Socorro María Rodríguez-Pinilla, Juana Serrano-López

**Affiliations:** 1 Pathology Department, Fundación Jiménez Díaz University Hospital, Madrid, Spain; 2 Department of Genetics, University of Cordoba, Maimonides Biomedical Research Institute of Cordoba (IMIBIC), Reina Sofía University Hospital, Córdoba, Spain; 3 Bioestatistics and Epidemiology Unit, Instituto de Investigación Sanitaria-Fundación Jiménez Díaz University Hospital, Madrid, Spain; 4 Immunology department, Fundación Jiménez Díaz University Hospital, UAM, Madrid, Spain; 5 Hematology Department, Fundación Jiménez Díaz University Hospital, Universidad Autónoma of Madrid (UAM), Madrid, Spain; 6 Hematology Department, Reina Sofía University Hospital/Maimonides Biomedical Research Institute of Córdoba (IMIBIC)/University of Córdoba, Córdoba, Spain; 7 Experimental Hematology Lab, Instituto de Investigación Sanitaria (IIS)-Fundación Jiménez Díaz, UAM, Madrid, Spain; 8 Centre for Haemato-Oncology, Barts Cancer Institute, Queen Mary University of London, London, United Kingdom; 9 Facultad de Biomedicina, Universidad Alfonso X el Sabio (UAX), Villanueva de la Cañada, Spain

**Keywords:** DLBCL, MAPK10, CCL18, RNA-seq, TME, LASSO

## Abstract

**Background:**

Activated B-cell (ABC) diffuse large B-cell lymphoma (DLBCL) has worse outcomes than the germinal center B-cell (GCB) subtype, but underlying molecular mechanisms remain poorly understood.

**Methods:**

Transcriptomic analysis on 43 DLBCL samples (23 GCB and 20 ABC) was performed using NanoString PanCancer Immune Profiling Panel with 30 cell-of-origin genes. Tumor microenvironment characterization was performed using CIBERSORTx and gene set enrichment analysis (GSEA) deconvolution. Based on our previous findings of MAPK10 downregulation in ABC lymphomas, MAPK10 promoter methylation was studied via pyrosequencing. Prognostic biomarkers were identified using the Cox regression and least absolute shrinkage and selection operator (LASSO) regularization. Therapeutic candidates were identified through connectivity mapping.

**Results:**

ABC lymphomas showed distinct profiles with the overexpression of *VTCN1*, *CDK4*, and *CXCR5* and the downregulation of *MMP9* and *MAPK10*. GSEA revealed enrichment of inflammatory pathways with immunosuppressive signals in ABC cases. Confirming our prior observations, *MAPK10* downregulation in ABC tumors was associated with promoter hypermethylation and inferior overall survival (*p* < 0.01). Immune deconvolution revealed greater microenvironmental diversity in ABC cases with significant eosinophil enrichment. High CD8^+^ T-cell abundance was associated with improved survival, particularly in ABC patients (*p* < 0.01). Multivariate analysis identified *CCL18* as an independent adverse prognostic factor (HR: 1.87, 95% CI: 1.25–2.79, *p* < 0.01). Connectivity mapping identified proteasome inhibitors and CDK4/6 inhibitors as promising therapeutic candidates.

**Conclusions:**

We validated *MAPK10* promoter hypermethylation and *CCL18* overexpression as prognostic biomarkers in ABC DLBCL. These findings, derived from integrative transcriptomic and immunogenomic profiling, provide clinically relevant insights into disease biology and support biomarker-guided strategies for precision treatment in aggressive B-cell lymphomas.

## 1 Introduction

Diffuse large B-cell lymphoma (DLBCL) represents the most common subtype of non-Hodgkin lymphoma (NHL), accounting for approximately 33%–40% of all lymphoma cases worldwide and constituting 4% of all cancer diagnoses globally. Incidence is higher in men than in women (1, 2). This malignancy is characterized by significant clinical, morphological, and molecular heterogeneity (3), which poses considerable diagnostic and therapeutic challenges. The majority of cases (80%) are classified as DLBCL not otherwise specified (DLBCL-NOS), while only 20% fall into specific DLBCL variants (4). The current standard of care for DLBCL-NOS involves the R-CHOP regimen (rituximab plus cyclophosphamide, hydroxydaunorubicin, oncovin, and prednisone), which has significantly improved patient outcomes with 5-year progression-free survival and overall survival rates of 60% and 65%, respectively (5–8). However, approximately 40% of patients experience relapse or refractory disease, facing a dismal prognosis with a median overall survival of only 6 months (9, 10). This clinical reality has driven the development of novel therapeutic approaches, including lenalidomide, bortezomib, ibrutinib, Chimeric Antigen Receptor (CAR) T-cell therapy, and bispecific antibodies (11, 12). Gene expression profiling has enabled the classification of DLBCL into molecularly distinct subtypes based on cell of origin (COO): germinal center B-cell (GCB)-like, activated B-cell (ABC)-like, and unclassified subtypes (13, 14). This molecular classification has demonstrated prognostic significance, with ABC DLBCL consistently associated with inferior outcomes compared to the GCB subtype (15, 16). Despite these molecular insights, current treatment protocols remain uniform across all subtypes, highlighting an unmet need for precision medicine approaches. The tumor microenvironment (TME) has emerged as a critical determinant of DLBCL biology and therapeutic response. Recent advances in single-cell RNA sequencing and spatial transcriptomics have revealed profound differences in immune landscapes between the GCB and ABC subtypes (17, 18). The ABC subtype typically exhibits an immunosuppressive microenvironment characterized by increased regulatory T cells, M2 macrophages, and enhanced PD-L1 expression, while GCB cases demonstrate a more immunoactive profile with higher CD8^+^ T-cell infiltration and interferon-γ signaling (9, 19). These microenvironmental distinctions directly correlate with differential responses to immunotherapies, particularly CAR T-cell therapy and immune checkpoint inhibitors, where GCB patients frequently achieve superior outcomes (20, 21). The spatial organization of immune cells within the TME, including the formation of tertiary lymphoid structures and the proximity of effector cells to malignant B cells, has emerged as a critical determinant of treatment efficacy (17, 18). Understanding these subtype-specific microenvironmental features is essential for developing tailored therapeutic strategies, as emerging evidence suggests that combination approaches targeting both tumor cells and their supportive niche may overcome the inherent chemoresistance observed in ABC DLBCL (22, 23). The integration of microenvironmental profiling with molecular subtyping represents a paradigm shift toward truly personalized lymphoma therapy, potentially transforming outcomes for patients with this heterogeneous malignancy. Herein, we performed gene expression analysis on 43 DLBCL samples using the NanoString PanCancer Immune Profiling Panel, customized with 30 COO genes for DLBCL subtyping. Comparative analysis between the ABC and GCB subtypes revealed differences in gene expression profiles, MAPK10 methylation patterns, pathway activation, immune cell infiltration, and clinical outcomes.

## 2 Patients and methods

### 2.1 Study design

This retrospective study was designed to investigate the microenvironmental differences between the GCB and ABC molecular subtypes of DLBCL. RNA specimens were derived from a previously established cohort described in our group’s prior publication (22), ensuring consistency with validated diagnostic criteria and sample quality standards. Sample collection, processing, and data were conducted through the IIS-Fundación Jiménez Díaz Biobank, following the technical and ethical standards established by the Spanish National Biobank Network. All samples underwent comprehensive anonymization protocols to ensure patient confidentiality and data protection in accordance with current privacy regulations.

The study cohort comprised 43 DLBCL cases selected retrospectively based on tissue availability and diagnostic quality, classified as GCB (n = 23) and ABC (n = 20) subtypes, based on the Lymphoma Subtyping Test (LST algorithm from NanoString) (23). Diagnostic specimens consisted of formalin-fixed, paraffin-embedded (FFPE) tumor biopsies obtained at the time of initial diagnosis, along with corresponding clinical and demographic data from participating institutions as detailed in our previous publication (22). To ensure diagnostic accuracy and consistency across the entire cohort, all cases underwent centralized pathological review by expert hematopathologists from the Pathology Department of Hospital Universitario Fundación Jiménez Díaz (HUFJD), who confirmed the original diagnoses and assessed tissue quality for molecular analysis. All study procedures were conducted in accordance with relevant clinical research guidelines and ethical standards, with informed consent obtained from all patients or their legal guardians prior to inclusion in the original study cohort.

All patients included in this study were adults, with a mean age of 65 years (range, 31–88 years). Sex distribution showed a slight male predominance (55.8%). Nearly half of the patients had an advanced stage of the disease, stage IV (48.8%) (24). Eastern Cooperative Oncology Group (ECOG) scale (25) had a balanced distribution across patients. Low-risk patients with International Prognostic Index (IPI) = 0–1 constituted the largest group with 20 patients (46.5%) (26). The vast majority of patients received standard immunochemotherapy with R-CHOP, accounting for 39 patients (90.7%). Alternative treatment regimens were used in a small minority, including rituximab, cyclophosphamide, vincristine, and prednisone (R-CVP) in one patient (2.3%), and other specialized regimens in three patients (7.0%). Regarding treatment response, 21 patients (48.8%) developed relapsed or refractory disease, and 16 patients (37.2%) died with a median follow-up of 20 months (range, 1–144 months) (see **Table 1**).

**Table 1:** Summary of characteristics of the patients.

Characteristics	Number (total N = 43)
Age (years)	
Mean (min–max)	65 (31–88)
Sex, N (%)	
Male	24 (55.8)
Female	19 (44.1)
Stage, N (%)	
I	3 (6.9)
II	11 (25.8)
III	8 (18.6)
IV	21 (48.8)
ECOG Performance Status, N (%)	
0	11 (25.8)
1	12 (27.9)
2	12 (27.9)
3	8 (18.6)
IPI-3, N (%)	
Low risk (0–1)	20 (46.5)
Intermediate risk (2–3)	11 (25.5)
High risk (4–5)	10 (23.2)
Missing data	2 (4.7)
Subtype, N (%)	
GCB	23 (53.5)
ABC	20 (46.5)
Treatment, N (%)	
R-CHOP	39 (90.6)
R-CVP	1 (2.3)
Other	3 (6.9)
Response to treatment, N (%)	
Refractory/relapsed (R/R)	21 (48.8)
Complete response (CR)	22 (51.1)
Survival status, N (%)	
Death	16 (37.2)
Follow-up (months)	
Median (min–max)	20 (1–144)

Stage was defined according to the Ann Arbor classification (24). ECOG Performance Status was assessed using the Eastern Cooperative Oncology Group criteria (25). Cell-of-origin subtype (GCB *vs*. ABC) was assigned using the Lymphoma Subtyping Test (LST) algorithm (23). Treatment categories (R-CHOP, R-CVP, and other) follow standard chemoimmunotherapy protocols for DLBCL. IPI risk groups were defined based on the International Prognostic Index (IPI) (26) 21.

GCB, germinal center B-cell-like; ABC, activated B-cell-like; R-CHOP, rituximab, cyclophosphamide, hydroxydaunorubicin, oncovin, and prednisone; R-CVP, rituximab, cyclophosphamide, vincristine, and prednisone; CR, complete response; R/R, refractory/relapsed.

### 2.2 RNA extraction and quality evaluation

Total mRNA was extracted from each sample using the RNeasy FFPE Kit (Qiagen GmbH, Hilden, Germany). Then, the concentration of each sample was measured on the NanoDrop 2000 (Thermo Fisher Scientific, Waltham, MA, USA). Quality control was evaluated using the DV300 parameter (% of RNA fragments greater than 300 bp) on the 4200 TapeStation system and the RNA ScreenTape kit (Agilent, Santa Clara, CA, USA).

### 2.3 NanoString analysis and data preparation

Gene expression (GE) profiling was performed using the nCounter^®^ Technology platform (NanoString Technologies, Seattle, WA, USA), a digital molecular barcoding system that enables direct quantification of mRNA transcripts without amplification. The NanoString nCounter^®^ PanCancer Immune Profiling Panel, which contains 750 predefined genes covering key immune pathways and cancer-related processes, was supplemented with 30 additional custom genes of interest (**Supplementary Table 1**), resulting in a comprehensive panel of up to 780 genes for analysis. These custom genes were specifically selected to enhance the detection of immune cell populations, cytokine signaling pathways, and tumor microenvironment markers relevant to the study objectives. The complete list of additional genes and their functional annotations can be found in **Supplementary Table 1**. Following data acquisition, the resulting raw count datasets underwent rigorous quality control assessment using nSolver Analysis Software v.4.0, which included the evaluation of binding density, imaging quality, and positive control linearity. Data normalization was subsequently performed using the geometric mean of housekeeping genes and positive control spike-ins to account for technical variation between samples, ensuring reliable and reproducible gene expression measurements across the entire dataset. To define differentially expressed genes (DEGs), the significance was determined using Benjamini–Hochberg adjusted *p*-values <0.05.

### 2.4 DNA methylation analysis

Genomic DNA was purified using QIAamp DNA FFPE Tissue Kit (Qiagen, Hilden, Germany), followed by bisulfite conversion using the EZ DNA Methylation-Gold Kit (Zymo Research, Irvine, CA, USA) according to the manufacturer’s instructions. A region of the *MAPK10* gene was amplified by PCR using Immolase DNA Polymerase (Bioline, London, UK) and bisulfite-specific primers: MAPK10_Fw1 (5′-GGGAATGGTTGAGTGATAGGA-3′), MAPK10_Rv1 (5′-CTCCAAAAACTTCCCCAAAACCTTCTAA, biotinylated-3′), and MAPK10_Seq1 (5-GGTTGAGTGATAGGAT-3′). The biotinylated PCR product was purified and converted to a single-stranded template using the Pyrosequencing Vacuum Prep Tool (Qiagen, Hilden, Germany). Pyrosequencing reactions were performed on the PyroMark Q24 system (Qiagen) according to the manufacturer’s guidelines. DNA methylation levels were quantified using the PyroMark Q24 software (Qiagen).

### 2.5 Deconvolution analysis

Several gene sets (GSs) were selected from different sources and databases to encompass all relevant populations and features. Enrichment scores for each GS and patient were computed independently using the GenePattern single-sample Gene Set Enrichment Analysis (ssGSEA) tool with the following parameters: 1,000 permutations for empirical significance testing, t-test as the ranking metric for differential expression, and gene set size filters ranging from a minimum of five genes to a maximum of 500 genes. False discovery rate (FDR) correction was applied using the Benjamini–Hochberg method. Only gene sets with FDR < 0.25 were considered significantly enriched. GE values for each sample were rank-normalized, and an enrichment score was produced using the Empirical Cumulative Distribution Functions of the genes in the GS and remaining genes. Normalized enrichment scores (NESs) were calculated by normalizing to the mean enrichment of random samples of the same size. The method employs random sampling of gene sets of the same size as the gene set being tested to assess significance and for normalization. Thus, a negative score means weaker relative activity in a sample compared with the background population, and a positive score means greater relative activity. In addition, normalized bulk GE data were used to infer the estimated proportions of infiltrating immune cells using the CIBERSORTx tool (https://cibersortx.stanford.edu/). CIBERSORTx GS were drawn from GE values of the LM22 predefined signature matrix (which evaluates the presence of 22 different human immune cell types) with some default parameters (Job type: impute cell fractions; signature matrix file: LM22; mixture file: GE dataset; permutations: 100; disable quantile normalization: TRUE; Batch correction: disabled; Run mode: relative).

### 2.6 Gene validation

Three complementary validation strategies were implemented to corroborate our findings using independent datasets. First, retrospective flow cytometry analysis of 20 DLBCL patients (GCB = 12 and non-GCB = 8) was conducted to quantify CD8^+^ T-cell infiltration frequencies between molecular subtypes, thereby validating our computational immune deconvolution results. Second, MAPK10 expression profiles were interrogated using the publicly available RNA-Seq repository Cancer Cell Line Encyclopedia (CCLE), with cell lines classified as GCB and high-grade subtypes. Third, the prognostic significance of both *MAPK10* and *CCL18* expression was assessed through survival analysis of 98 samples from the RNA-sequencing dataset with corresponding clinical metadata generated by Schmitz et al. (27) (Genetics and Pathogenesis of Diffuse Large B Cell Lymphoma | NCI Genomic Data Commons). *CCL18* expression across normal and tumor tissues was examined using GEPIA 2 (GEPIA 2).

### 2.7 Connectivity mapping

Reverse transcriptomic analysis was conducted using the Integrative Library of Integrated Network-Based Cellular Signatures (iLINCS) platform (28), as described previously (29). A query of the full ABC *vs*. GCB signature comprising DEGs was generated from the comparison. This signature was submitted to iLINCS for reverse transcriptomic connectivity analysis against LINCS chemical perturbagen transcriptional profiles (143,374 signatures; accessed on 14 June 2025). Chemical perturbagens were considered significant if they exhibited a Benjamini–Hochberg adjusted FDR < 0.01.

### 2.8 Statistical and survival analyses

GE data from the panel, enrichment score from ssGSEA, and cell population frequency from CIBERSORTx were analyzed independently using pipelines developed in Rv4.2.1 (The R Foundation for Statistical Computing, Vienna, Austria). A Shapiro–Wilk normality test was used before comparing means or medians. Then, significance (*p* < 0.05) was determined using Student’s unpaired samples t-tests when variables were normally distributed, and Kruskal–Wallis non-parametric tests otherwise. Variables demonstrating statistical significance with *p* < 0.05 in the univariate analysis for the whole cohort were selected as candidates for inclusion in the multivariate model. The least absolute shrinkage and selection operator (LASSO) regularization technique was then employed for optimal variable selection (GE and infiltrated immune cells), enabling the construction of robust multivariate predictive models for overall survival (OS) and disease progression outcomes. The LASSO regularization path and optimal penalty parameter (λ) were determined using leave-one-out (LOO) cross-validation. Model selection followed the “one standard error rule” (λ_1se), which selects the most parsimonious model within one standard error of the minimum cross-validation error. The regularization path (coefficient trajectories) and cross-validation error curve are provided in **Supplementary Figures 6A and 6B**, respectively. The Kaplan–Meier method was used to estimate patient survival, and groups’ survival curves were compared using the log-rank test. Graphs were generated using GraphPad Prism 8 (GraphPad Software Inc., La Jolla, CA, USA).

## 3 Results

### 3.1 Tumor microenvironment signature of ABC lymphomas

**Figure 1:**
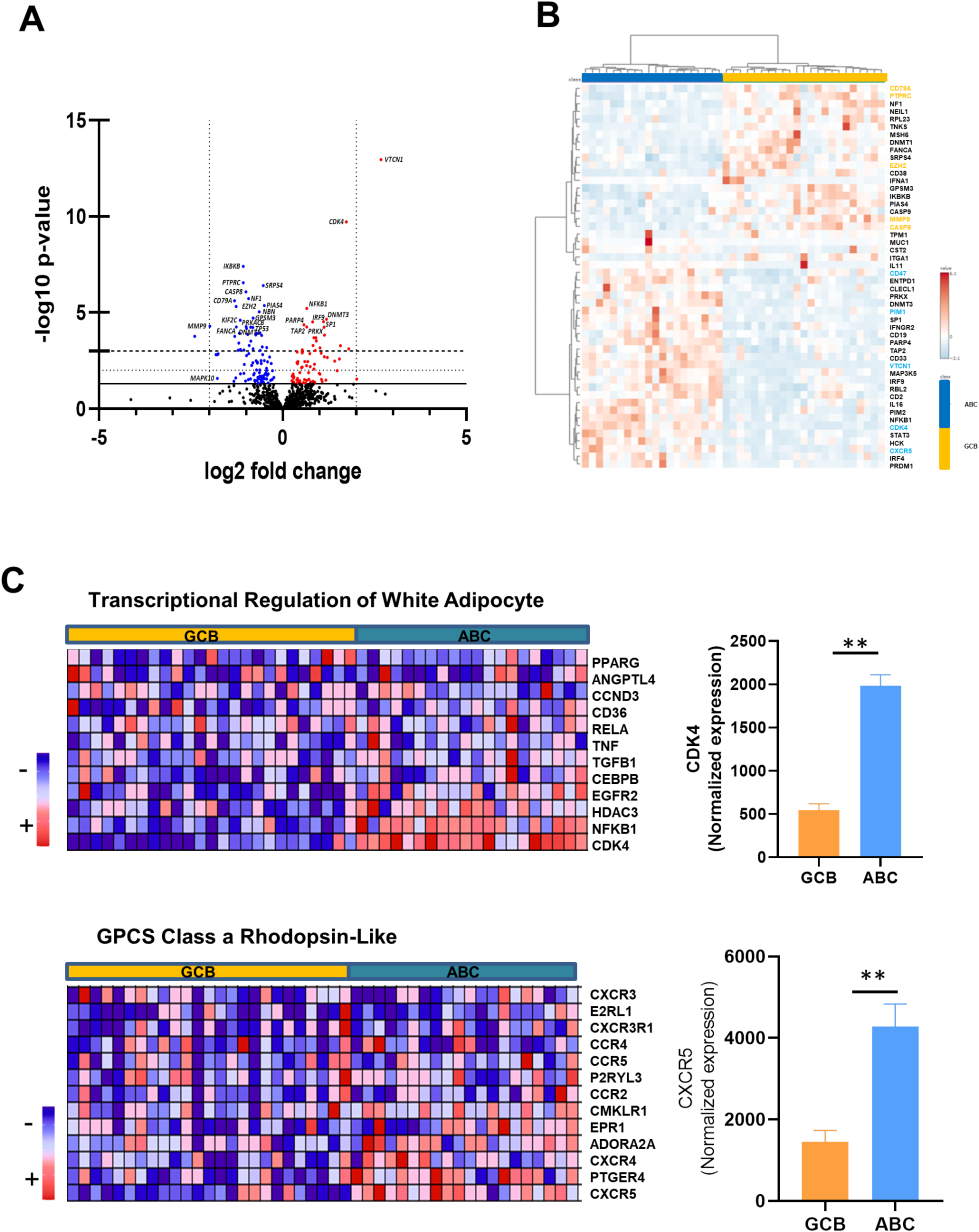
Differential expression of genes in DLBCL subtypes. **(A)** Volcano plot showing differentially expressed genes between ABC and GCB subtypes (n = 23 each). Genes on the right side are upregulated in the ABC group, while those on the left are relatively overexpressed in the GCB group. Threshold lines indicate statistical significance: solid line for *p* < 0.05, dotted line for *p* < 0.01, and dashed line for *p* < 0.001. **(B)** Heatmap displaying the expression of the top 50 differentially expressed genes across ABC and GCB DLBCL subtypes. Colored genes represent features with significant differences between groups (t-test, *p* < 0.01). **(C)** Heatmaps summarizing gene set enrichment analysis (GSEA) results for two pathways significantly enriched (FDR < 0.05). Enrichment plots are shown in the top and bottom left panels. The bar plots on the right display the normalized NanoString expression values of representative genes CDK4 (top) and CXCR5 (bottom), both of which were significantly upregulated in the ABC subtype. **p < 0.01. DLBCL, diffuse large B-cell lymphoma; ABC, activated B cell; GCB, germinal center B cell; FDR, false discovery rate.

To visualize DEGs distinguishing the ABC subtype from the GCB subtype, we generated a volcano plot (**Figure 1A**) and a heatmap (**Figure 1B**). This scatter plot shows a total of 174 DEGs, where two top significant upregulated genes in the ABC lymphomas (**Figure 1A**, top right, *p* < 0.05), corresponding to *VTCN1* [>2 fold change (FC)] and *CDK4* (>1.5 FC). *MMP9* (>−2 FC) was markedly downregulated in this type of lymphoma compared with the GCB class (**Figure 1A**, left). Other genes, such as *IKBKB*, *PTPRC*, *CASP8*, or *CD79B*, with small *p*-values did not have a great magnitude of changes between the lymphoma subtypes. Hierarchical clustering of the top 50 genes in the heatmap revealed two gene clusters that clearly separate the DLBCL subtypes. Cluster 1 (**Figure 1B**, top right) included genes such as *CD79A*, *PTPRC*, *EZH2*, *MMP9*, *IKBKB*, or *CASP8*, which were upregulated in GCB lymphomas (**Figure 1B**, top right colored in orange). These genes showed statistically significant differences between subtypes (t-test, *p* < 0.01). In contrast, cluster 2 comprised ABC upregulated genes such as *CD47*, *PIM1*, *VTCN1*, *CDK4*, and *CXCR5* (**Figure 1B**, bottom left colored in blue), also demonstrating significant separation between groups.

To further explore the biological pathways associated with our DEGs, we performed ssGSEA. We focused on gene sets with an FDR < 0.25, in line with the standard threshold for GSEA significance. This FDR threshold included five pathways listed in **Supplementary Figure 1A** and named TGF-β receptor signaling in skeletal disease, transcriptional regulation of white adipocyte (TRWA), TGF-β receptor signaling, TNF-α, and GPCRS, Class A Rhodopsin-Like (GCRL). All of them had similar negative NES values, indicating a bottom enrichment of gene set for the GCB subtype (**Supplementary Figure 1A**). Among these signaling pathways, we focused on two of them (**Figure 1C**), as they contain relevant genes that may be involved in the pathogenesis of the ABC lymphomas. Interestingly, the TRWA pathway showed that the *CDK4* gene was significantly overexpressed in the ABC lymphomas (**Figure 1C**, top right), indicating an increase in cell cycle or elevated proliferation rates. The second pathway selected was GCRL, showing levels increased for *CXCR5* in ABC samples (**Figure 1C**, bottom right), suggesting an enrichment of follicular helper T cell (Tfh) signature. In this sense, deep analysis determined that the *PDCD1* gene was also increased in the ABC lymphomas (**Supplementary Figure 1B**). Finally, signaling pathways such as TNF-α and TGF-β receptor signaling further support the overexpression of NF-κB in the ABC subtype (**Supplementary Figure 1C**). These results support the biological differences between subtypes, with ABC displaying transcriptional programs related to proliferation and immune signaling.

Altogether, transcriptomic and pathway analyses revealed that ABC lymphomas are characterized by the upregulation of genes involved in immune evasion, cell cycle progression, and Tfh cell-associated signaling, underscoring a transcriptional program consistent with enhanced proliferation and immune modulation compared with the GCB lymphomas.

### 3.2 MAPK10 downregulation in ABC subtype is associated with methylation-driven silencing and adverse outcomes

**Figure 2:**
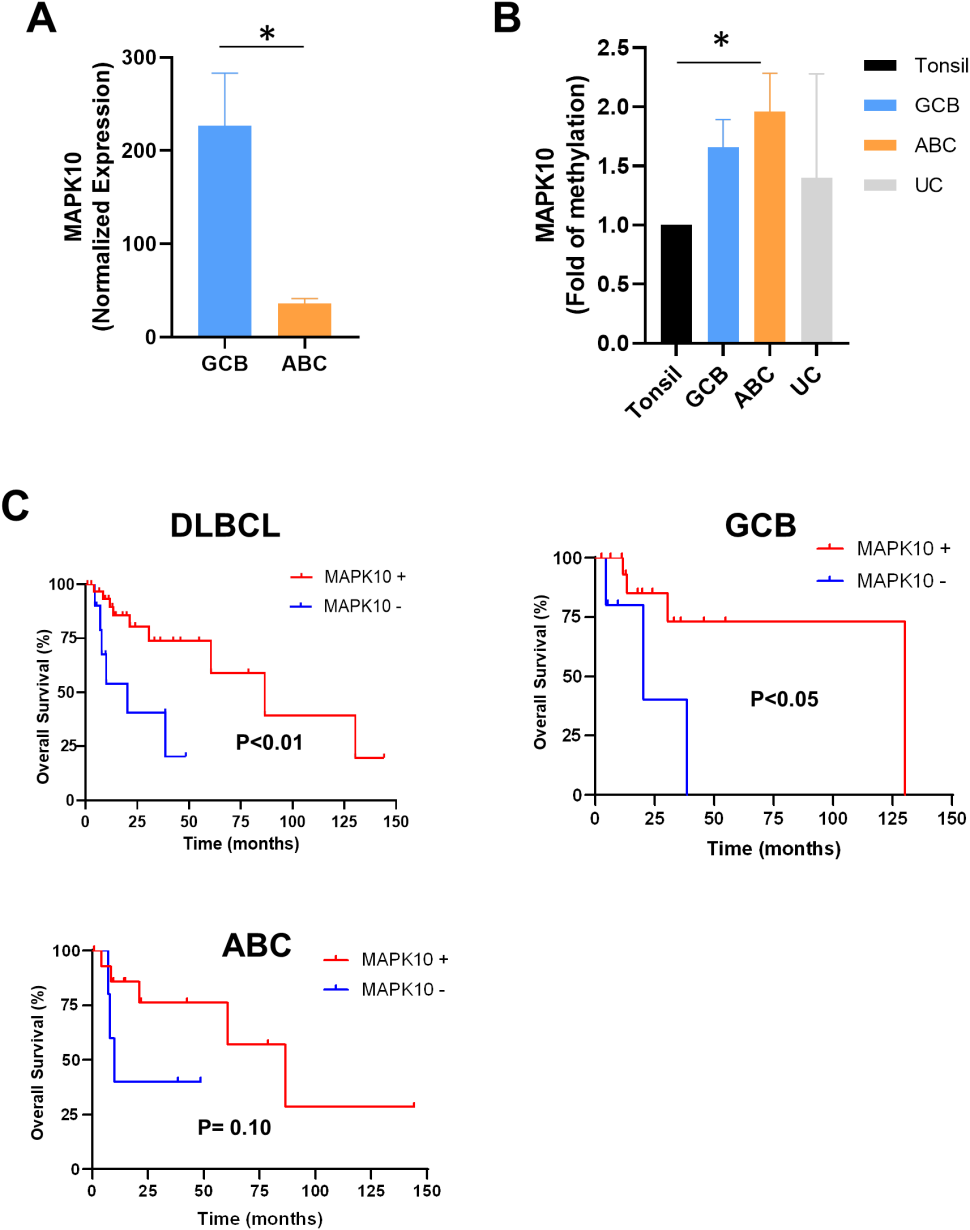
*MAPK10* expression levels and prognostic significance across DLBCL subtypes. **(A)** Comparison of normalized NanoString expression values of *MAPK10* between GCB and ABC subtypes of DLBCL, showing significantly higher expression in GCB subtype. **(B)***MAPK10* methylation distribution across tissue types including normal tonsil, GCB-DLBCL, ABC-DLBCL, and UC-DLBCL, showing significantly elevated methyletion profile in the ABC compared with normal tonsil. **(C)** Kaplan-Meier overall survival curves stratified by *MAPK10* expression status for all DLBCL cases (left panel), GCB subtype (right panel), and ABC subtype (bottom-left panel). Statistical significance assessed using log-rank test. MAPK10 positivity confers significantly improved overall survival specifically in whole DLBCL and in the GCB subtype, with no significant association observed in ABC subtype or the combined DLBCL cohort. Statistical comparisons performed using log-rank test, with p-values indicated in each panel. Bar graph data presented as mean ± SEM. * *p*<0.05.

We previously described that the *MAPK10* tumor suppressor gene is downregulated in the ABC lymphomas (29). Analysis of *MAPK10* expression in this new cohort of DLBCL patients also revealed a marked downregulation in the ABC subtype compared to the GCB subtype (**Figure 2A**, top left), confirming our previous findings. Moreover, similar significant differences were found in high-grade lymphoma cell lines compared to GCB (**Supplementary Figure 2A**).

DNA methylation, particularly in promoter regions, is a well-established epigenetic mechanism associated with transcriptional repression. To explore whether this mechanism could explain the downregulation of *MAPK10* observed in ABC lymphomas, we performed pyrosequencing to quantify methylation levels in its promoter region. We analyzed a total of 21 CpG sites within the *MAPK10* promoter region, and we calculated the mean methylation percentage for each group of samples to provide an overall measure of promoter methylation. As a result, the ABC group (N = 10) exhibited a significantly higher average level of promoter methylation than non-malignant tonsillar tissues (N = 10), with a modest increase when compared to the GCB group (N = 10) (**Figure 2B**). To further investigate clinical implications, we assessed the *MAPK10* downregulation in DLBCL on OS using Kaplan–Meier survival analysis. Our findings suggest that patients with a higher level of *MAPK10* gene expression (over 25th percentile) had a significant increase in OS (**Figure 2C**) in the whole cohort (**Figure 2C**, top left) (mean ± SE: 84.6 ± 14.4 *vs*. 23.8 ± 6.0, *p* < 0.01) and in the GCB group (**Figure 2C**, top right) (mean ± SE: 100.6 ± 17.4 *vs*. 24.4 ± 8.3, *p* < 0.05). We observed a trend toward shorter OS in the ABC group (**Figure 2C**, bottom) (mean ± SE: 80.4 ± 18.2 *vs*. 24.4 ± 8.8, *p* < 0.10) without statistical significance. Median follow-up was 20 months (0–144 months). These findings suggest that *MAPK10* gene expression is epigenetically silenced through promoter hypermethylation in primary DLBCL samples, particularly within the ABC subtype, supporting its role as a tumor suppressor whose loss contributes to adverse clinical outcomes.

### 3.3 Cell type deconvolution analysis revealed high transcriptomic diversity in ABC lymphomas

**Figure 3:**
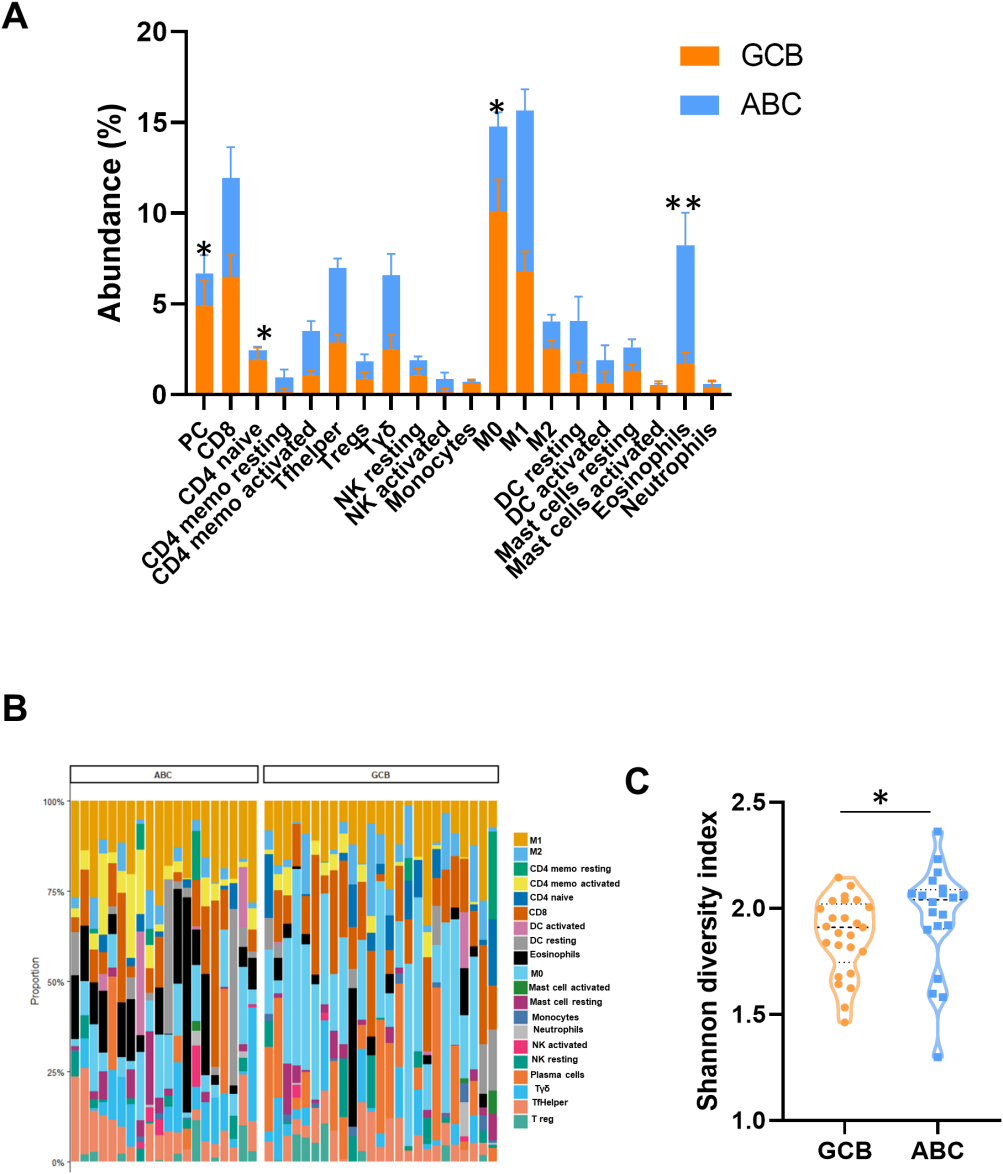
Tumor microenvironment immune cell composition and diversity in DLBCL subtypes. **(A)** Stack bars of relative abundance of immune cell populations estimated by CIBERSORTx in GCB (orange color) and ABC (blue color) DLBCL subtypes, as determined by CIBERSORTx deconvolution analysis. Bar plots display mean proportions with error bars representing variability across samples. Asterisks indicate statistically significant differences between subtypes. **(B)** Immune cell composition estimated by CIBERSORTx in ABC and GCB DLBCL samples. Stacked bar plots show the relative proportions of 22 immune cell subsets for each sample, highlighting differences in the tumor microenvironment between ABC and GCB subtypes. **(C)** Shannon diversity index quantifying immune cell composition heterogeneity within each DLBCL subtype. Violin plot demonstrates significantly greater immune diversity in GCB compared to ABC subtype. *p < 0.05, **p < 0.01. PC, plasma cells; CD4 memo, CD4 memory; Tfh, follicular helper T cells; Tregs, regulatory T cells; Tγδ, gamma delta T cells; DC, dendritic cells; DLBCL, diffuse large B-cell lymphoma; GCB, germinal center B cell; ABC, activated B cell.

To assess the immune cell composition of the tumor microenvironment of the DLBCL subtypes, we applied CIBERSORTx to our customized PanCancer IO 360-derived transcriptomic data. For downstream visualization and comparative analyses, we excluded memory and naïve B-cell populations in order to focus specifically on non-tumoral immune cell components. Thus, ABC tumors were significantly enriched in eosinophils, while GCB samples showed higher abundance of plasma cells and CD4-naïve T cells (**Figures 3A, B**). Then, we wanted to know the entropy level in each subtype of lymphoma. To address this, we calculated the Shannon diversity index for each DLBCL subtype to analyze immune diversity. ABC samples exhibited significantly higher microenvironmental diversity compared to GCB samples, suggesting a more heterogeneous immune infiltrate (**Figure 3C**). Importantly, ABC cases exhibited an enrichment of Tfh cell signatures (*p* = 0.06), consistent with the elevated gene expression of *CXCR5* and *PDCD1* previously mentioned (**Figure 1C**, **Supplementary Figure 2B**). These findings highlight distinct immune niches associated with each DLBCL subtype. Moreover, using the CIBERSORTx-derived cell fraction matrix, we conducted orthogonal partial least squares discriminant analysis (OPLS-DA) to build a classification model capable of discriminating between the ABC and GCB subtypes (**Supplementary Figure 3A**). This analysis identified plasma cells, eosinophils, CD4^+^ memory activated, and CD8^+^ T cells as the most influential contributors to group separation, as reflected by the higher Variable Importance in Projection (VIP) scores (**Supplementary Figure 3B**). Notably, we further validated an enrichment of CD8^+^ T cells in non-GCB specimens using flow cytometry analysis of an independent cohort of lymph node samples (**Supplementary Figure 3C**), providing orthogonal confirmation of our transcriptomic findings and supporting the biological relevance of the observed microenvironmental differences between the DLBCL subtypes. In summary, immune cell deconvolution and diversity analysis revealed that ABC lymphomas display a highly heterogeneous and immunologically complex microenvironment characterized by eosinophils and Tfh enrichment, supporting the existence of distinct immune niches that differentiate the ABC subtype from the GCB subtype.

### 3.4 Clinical impact of immune cell abundance and gene expression profile

**Figure 4:**
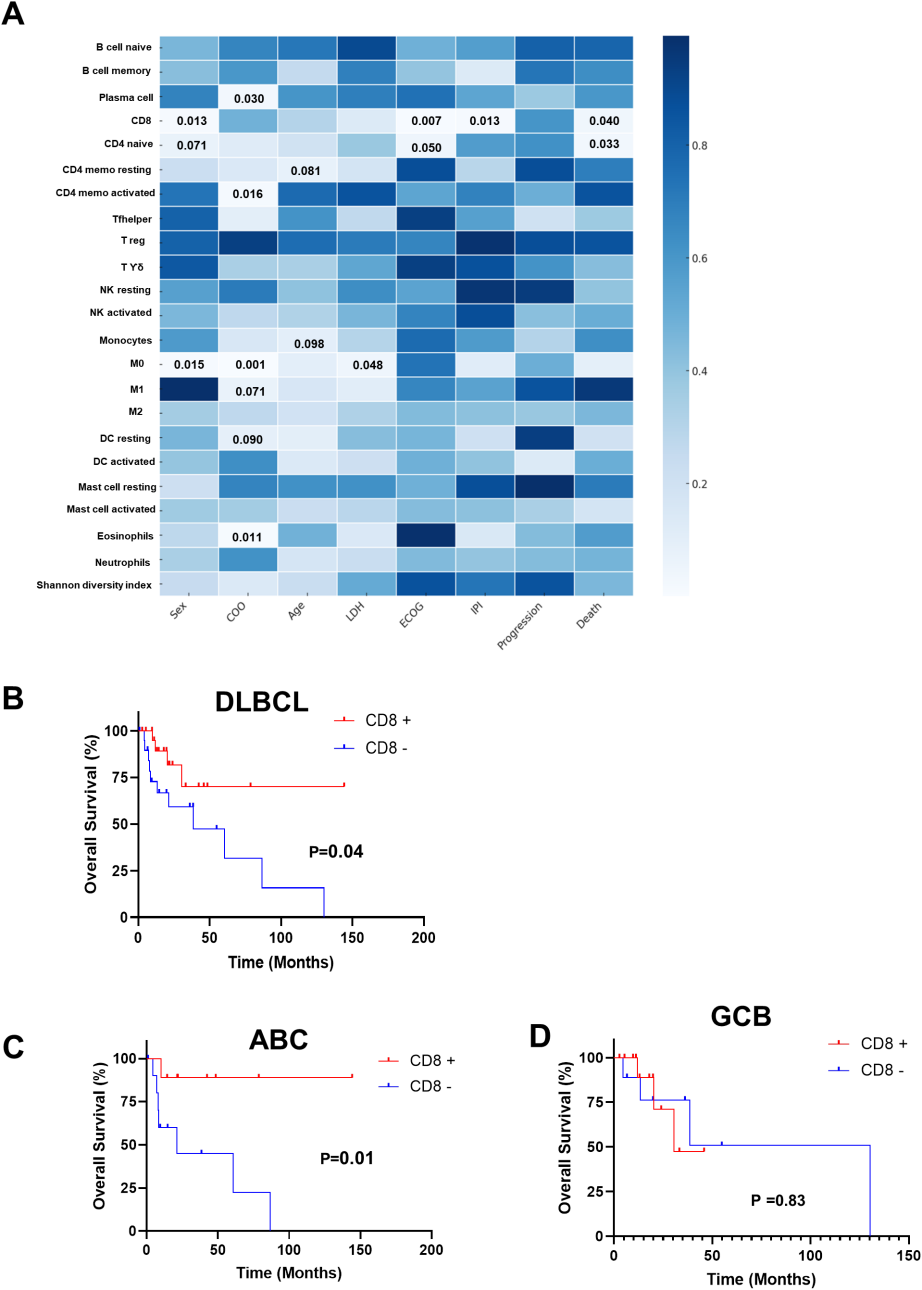
Immune microenvironment associations and prognostic impact in DLBCL subtypes. **(A)** Heatmap of statistical significance (p-values) from t-test comparisons of immune cell proportions between CD8-high (CD8^+^) and CD8-low (CD8^−^) cases across DLBCL subtypes. Color intensity corresponds to degree of statistical significance, with darker shading indicating lower p-values. Only p < 0.1 are annotated within the heatmap. **(B–D)** Kaplan–Meier overall survival analysis stratified by CD8 expression status for the complete DLBCL cohort **(B)**, GCB subtype **(C)**, and ABC subtype **(D)**. CD8+ infiltration confers significantly improved survival in the overall DLBCL cohort and ABC subtype, with no significant association in GCB subtype. Statistical comparisons performed using log-rank test. CD4 memo, CD4 memory; Tfh, T follicular helper; Tregs, regulatory T cells; Tϒδ, gamma delta T cells; DC, dendritic cells; DLBCL, diffuse large B-cell lymphoma; GCB, germinal center B cell; ABC, activated B cell.

To investigate associations between tumor immune composition and clinical features, the mean abundance of CIBERSORT-inferred immune cell subsets was compared across key clinical variables using Student’s t-test. The resulting *p*-values are visualized as a heatmap in **Figure 4A**. Significant differences were observed for several cell populations. Of note, CD8^+^ T-cell abundance was reduced in male patients (mean ± SE: 0.035 ± 0.009 *vs*. 0.083 ± 0.01, *p* < 0.05), patients with elevated ECOG Performance Status (mean ± SE: 0.027 ± 0.007 *vs*. 0.083 ± 0.014, *p* < 0.01), patients with higher IPI score (mean ± SE: 0.032 ± 0.006 *vs*. 0.081 ± 0.01, *p* < 0.05), and patients who died during follow-up (mean ± SE: 0.034 ± 0.01 *vs*. 0.076 ± 0.01, *p* < 0.05). Furthermore, to evaluate the prognostic significance of CD8^+^ T-cell abundance, Kaplan–Meier survival analyses were conducted. Median follow-up was 20 months (range, 0–144 months). The median value was used as the cutoff point. High CIBERSORT-derived CD8^+^ T-cell abundance was significantly associated with improved overall survival in the DLBCL cohort, with a mean ± SE of 107.32 ± 16.45 *vs*. 52.57 ± 13.62 (*p* < 0.01; **Figure 4B**). Upon stratification by molecular subtype, this survival advantage remained significant in patients with the ABC subtype (mean ± SE: 129.32 ± 14.06 *vs*. 39.10 ± 12.42, *p* < 0.04; **Figure 4C**), whereas no significant association was observed in the GCB subgroup (mean ± SE: 33.94 ± 5.11 *vs*. 78.18 *vs*. 26.83, *p* = 0.83; **Figure 4D**). These findings suggest that reduced cytotoxic T-cell infiltration is associated with adverse clinical outcomes, positioning CD8^+^ T cells as a potentially protective immune component in ABC DLBCL. They also support the relevance of CD8^+^ T cells as prognostic biomarkers (30).

Finally, Cox univariate analysis was conducted to assess the prognostic significance of individual biological features on global survival in a COO-dependent manner. Biological features included gene expression features (N = 780) and CIBERSORT-inferred immune cell subsets (N = 44). First, a list of features was initially selected from the whole DLBCL cohort based on a univariate *p*-value threshold of less than 0.05. Each selected variable was then independently analyzed using the Cox proportional hazards model to evaluate its association with overall patient survival (**Supplementary Table 2**). This approach enabled the identification of potential biomarkers with differential prognostic relevance across the COO subtypes (**Supplementary Table 2**).

In further multivariate modeling, LASSO regression identified *CCL18* as the only feature independently associated with a significantly adverse impact on overall survival (*p* < 0.01), with a hazard ratio (HR) of 1.87 (95% CI: 1.25–2.79) and a C-statistic of 0.81 (**Supplementary Table 3**). Comparative tumor analysis of *CCL18* expression in GEPIA 2 (GEPIA 2) demonstrated significantly elevated expression levels in DLBCL relative to other malignant tumor types (**Supplementary Figure 4A**). Cell type-specific expression profiling revealed that immunosuppressive M2-polarized macrophages exhibit the highest *CCL18* expression levels among all infiltrating monocyte and macrophage populations (**Supplementary Figure 4B**).

**Figure 5:**
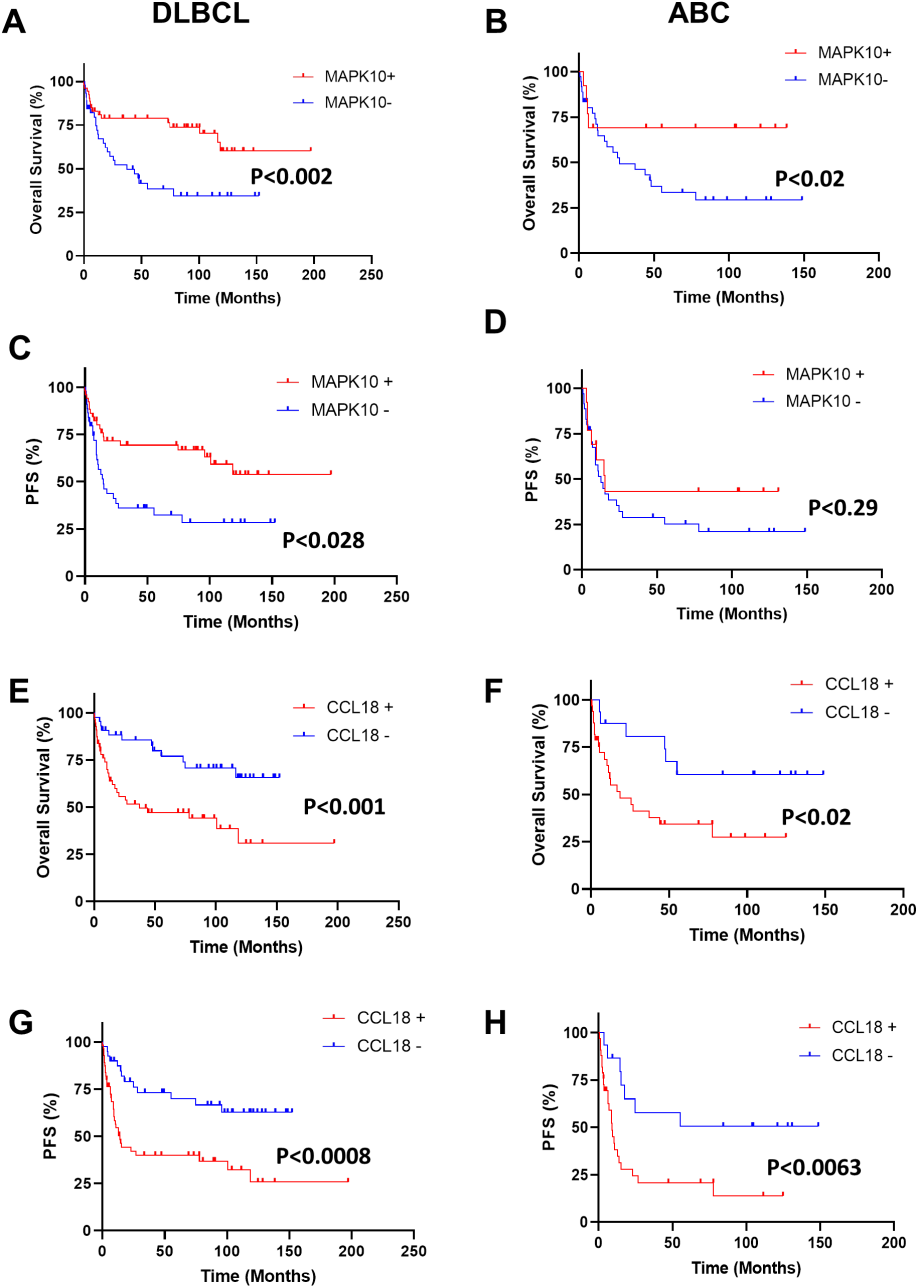
Prognostic impact of tumor microenvironment-related gene expression in the validation cohort of DLBCL patients. Kaplan–Meier survival analyses were performed in an independent validation cohort of 98 patients (49 GCB and 49 ABC). **(A–H)**. Curves show overall survival (OS) and progression-free survival (PFS) according to MAPK10 **(A–D)** and CCL18 gene expression **(E–H)**, stratified into high (red) and low (blue) expression groups. Left panels correspond to the whole DLBCL cohort, while right panels show subgroup analyses restricted to the ABC subtype. Statistical differences between groups were calculated using the log-rank test, with p-values indicated in each panel. DLBCL, diffuse large B-cell lymphoma; GCB, germinal center B cell; ABC, activated B cell.

Finally, in the independent RNA-seq validation cohort of 98 patients with DLBCL, the prognostic relevance of *MAPK10* and *CCL18* expression was evaluated using Kaplan–Meier survival analysis (**Figure 5**). Patients with high *MAPK10* expression exhibited significantly inferior OS compared to MAPK10-low cases (*p* = 0.002; **Figure 5A**). This adverse effect was particularly evident in the ABC-like subtype (*p* = 0.02; **Figure 5B**), while no significant difference was observed in GCB-like tumors (**Supplementary Figure 4C**). Consistently, high *MAPK10* levels were also associated with shorter progression-free survival (PFS) in the overall cohort (*p* = 0.028; **Figure 5C**), although this trend did not reach statistical significance when restricted to ABC cases (*p* = 0.29; **Figure 5D**) or GCB cases (**Supplementary Figure 4D**). Similarly, elevated *CCL18* expression identified patients with significantly reduced OS (*p* = 0.001; **Figure 5E**) and PFS (*p* = 0.0008; **Figure 5G**) across the entire cohort. Stratified analyses revealed that this prognostic effect was largely driven by ABC DLBCL, where high *CCL18* expression correlated with markedly worse OS (*p* = 0.02; **Figure 5F**) and PFS (*p* = 0.0063; **Figure 5H**) without differences in GCB (**Supplementary Figure 4E, 5F**). Together, these results demonstrate that both *MAPK10* and *CCL18* overexpression are linked to unfavorable clinical outcomes in DLBCL, with the adverse prognostic effect of *CCL18* being particularly pronounced in the ABC subtype.

Consistent with our previous findings (**Figure 3A**), additional validation analyses showed a trend toward eosinophil enrichment (*p* = 0.23) and CD4 memory activated (*p* < 0.01) in the ABC subtype, and a significant enrichment of M0 macrophages in the GCB subtype (**Supplementary Figure 5A**). Correlation analyses revealed no strong associations among *CCL18*, *MAPK10*, and the CD8 score across DLBCL as a whole. The highest correlation observed was the negative association between *CCL18* and *MAPK10* (r = −0.31), which was similarly detected in both DLBCL and the ABC subset, whereas CD8 scores showed no meaningful correlation with either marker (**Supplementary Figure 5B**). Finally, to further dissect the interaction between cytotoxic infiltration, *CCL18*, and *MAPK10*, patients were stratified according to high versus low CD8 infiltration in the CCL18-positive group, and survival within the MAPK10^−^ and MAPK10^+^ subsets was examined. Although there were no significant differences in the CCL18^+^/MAPK10^−^ subgroup, high CD8 infiltration did not confer any survival benefit (**Supplementary Figure 5C**). However, in the CCL18^+^/MAPK10^+^ subgroup, the pattern reversed: patients with high CD8 infiltration showed a tendency toward improved survival compared with those with low CD8 infiltration (**Supplementary Figure 5D**). These findings suggest that, in the absence of MAPK10, the adverse influence of CCL18 dominates, whereas MAPK10 positivity appears to restore the favorable prognostic value of cytotoxic T-cell infiltration.

Together, these analyses identify CD8^+^ T cells as a protective immune component and *CCL18* as an independent adverse prognostic factor in ABC DLBCL, with both findings validated in an independent patient cohort, supporting their potential clinical utility for risk stratification.

### 3.5 Therapeutic candidates for ABC lymphoma through connectivity mapping

**Figure 6:**
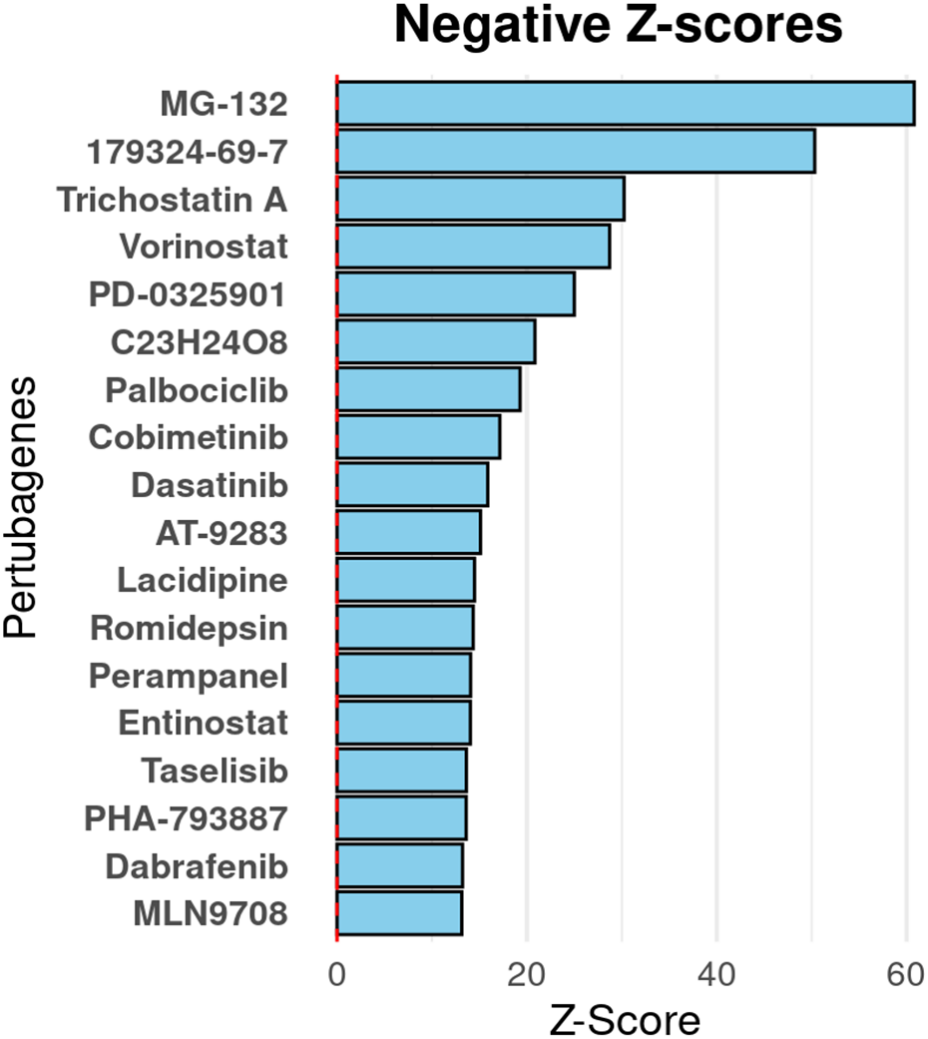
iLINCS connectivity analysis identifies small-molecule perturbagens predicted to reverse ABC DLBCL transcriptome toward GCB-like expression profile. Bar graph displaying top-ranked small-molecule perturbagens based on negative connectivity Z-scores, indicating inverse correlation with the ABC-specific gene expression signature relative to GCB DLBCL. More negative Z-scores reflect greater predicted capacity to transcriptionally reprogram ABC-like cells toward a GCB-like phenotype. Notable top-ranking compounds include MG-132 (a proteasome inhibitor that disrupts protein degradation and inhibits NF-κB signaling to promote apoptosis in ABC DLBCL) and 179324-69-7 (an HSP90 inhibitor that induces degradation of oncogenic client proteins critical for ABC cell survival). These results suggest therapeutic potential for targeting ABC-specific oncogenic pathways through proteasome and HSP90 inhibition. iLINCS, Integrative Library of Integrated Network-Based Cellular Signatures; ABC, activated B cell; DLBCL, diffuse large B-cell lymphoma; GCB, germinal center B cell.

To identify potential therapeutic compounds capable of reverting the ABC oncotranscriptome toward a less aggressive disease phenotype, we conducted an integrative computational analysis using the iLINCS. This approach systematically evaluated drug-induced transcriptomic perturbations to identify compounds with negative connectivity scores, which indicate potential for reversing the ABC-specific gene expression signature (**Figure 6**, **Supplementary Table 4**). The analysis ranked compounds based on their anti-correlation strength with the ABC lymphoma transcriptomic profile, prioritizing those most likely to counteract the molecular drivers of this aggressive subtype. Among the highest-ranking candidates were MG-132 and 179324-69-7 (bortezomib), both established proteasome inhibitors that demonstrated strong negative connectivity scores, suggesting robust potential for transcriptomic reversion of the ABC phenotype. These findings are particularly noteworthy given the established clinical efficacy of proteasome inhibition in aggressive B-cell lymphomas. Additionally, palbociclib, a selective CDK4/6 inhibitor, emerged as a compound of interest through complementary volcano plot analysis, where it was associated with the downregulation of highly expressed genes characteristic of the ABC subtype, despite exhibiting a more moderate overall connectivity score compared to the proteasome inhibitors. This integrative computational approach successfully identified mechanistically relevant therapeutic candidates, proteasome inhibitors targeting NF-κB-driven ABC pathogenesis, and CDK4/6 inhibitors addressing the observed *CDK4* overexpression, which represent promising starting points for experimental validation and potential combination therapy strategies in ABC DLBCL.

## 4 Discussion

Our comprehensive transcriptomic and immunogenomic analyses reveal distinct molecular and microenvironmental features that differentiate the ABC subtype of DLBCL from its GCB counterpart. These findings not only reinforce previously described subtype-specific hallmarks but also identify novel transcriptional and epigenetic targets with potential prognostic and therapeutic implications. The overexpression of genes such as *VTCN1*, *CDK4*, and *CXCR5* in ABC DLBCL highlights a transcriptional program enriched in immune evasion, cell cycle progression, and Tfh cell recruitment, respectively. *VTCN1* (also known as B7-H4), a negative regulator of T-cell activity, is known to contribute to T-cell anergy and poor antigen presentation, suggesting a mechanism of immune suppression in ABC tumors (31). Consistent with this, FOXP1 overexpression, a defining feature of the ABC subtype, has been shown to suppress immune response signature and MHC class II expression, further contributing to impaired antigen presentation and T-cell activation (32), while CD58 alterations have been reported to induce PD-L1 and IDO expression, further promoting immune evasion in DLBCL (33). Similarly, *CDK4* overexpression aligns with increased proliferative capacity, and its role in lymphomagenesis is supported by studies demonstrating the sensitivity of CDK4/6-driven malignancies to pharmacologic inhibition and poor prognosis in DLBCL and other B-cell lymphomas (34).

The upregulation of *CXCR5* and *PDCD1* (PD-1) suggests Tfh enrichment, corroborated by immune deconvolution analysis. This pattern reflects an immunologically active yet potentially exhausted microenvironment, a hallmark of chronic inflammatory states. Notably, anti-CXCR5 therapies based on CAR-T therapies have been shown to more effectively eliminate B-NHL cells and their supportive Tfh cells compared to conventional CD19-directed CAR T cells (35). Our findings underscore the importance of a better understanding of the TME to inform and optimize the design of next-generation CAR T-cell therapies.

Gene set enrichment analysis (GSEA) further identified significant enrichment of inflammatory and signaling pathways, including transcriptional regulation of white adipocyte, TNF-α, TGF-β receptor, and GPCR-related pathways, all more prominently activated in ABC cases. These pathways are intricately linked to NF-κB signaling, a central driver of ABC pathogenesis (36–38). These data provide a molecular rationale for the therapeutic targeting of these signaling axes in ABC DLBCL. Given their roles in promoting pro-tumor inflammation, tumor progression, and cell migration and trafficking, combining pathway-specific inhibitors with next-generation CAR T-cell therapy or BTK inhibitors may offer enhanced therapeutic efficacy in ABC cases characterized by the activation of multiple signaling pathways.

A key finding of our study is the epigenetic silencing of *MAPK10* through promoter hypermethylation in ABC tumors. *MAPK10* (also known as JNK3) has previously been characterized as a tumor suppressor gene across various cancers, playing critical roles in apoptotic signaling and stress response (39). In hepatocellular carcinoma, *MAPK10* serves as a prognostic marker of the immunosuppressive tumor microenvironment, where its downregulation correlates with diminished overall survival (40). Our group previously reported decreased *MAPK10* expression levels in ABC lymphomas (29). The present study corroborates these findings and, importantly, demonstrates that this silencing correlates with significant hypermethylation of the *MAPK10* promoter region specifically in the ABC subtype. These observations align with similar epigenetic alterations reported in DLBCL cell lines (41). Because promoter CpG methylation of *MAPK10* showed only marginal subtype differences, several additional mechanisms may contribute to its reduced expression in ABC DLBCL. These include altered chromatin accessibility or methylation at distal regulatory elements, repressive histone modifications, post-transcriptional regulation by microRNAs or long non-coding RNAs, and genomic alterations affecting *MAPK10* or its regulatory context. Future studies incorporating Assay for Transposase-Accessible Chromatin using Sequencing (ATAC-seq), histone-mark profiling, and small RNA analyses are needed to clarify these regulatory layers.

Furthermore, our survival analysis revealed that reduced *MAPK10* expression in DLBCL is associated with inferior overall survival, particularly pronounced within the ABC subtype (**Figures 2C**, **5B**), positioning *MAPK10* as both a potential prognostic biomarker and a promising target for epigenetic therapeutic intervention in the ABC lymphomas.

CIBERSORTx-based immune deconvolution revealed greater immune diversity in ABC DLBCL, characterized by a notable enrichment of eosinophils. The role of eosinophils within the TME remains unclear. Both pro- and anti-tumorigenic responses have been attributed to TME-infiltrating eosinophils, depending on the cytokine milieu that shapes the Th1/Th2 balance (42). Interestingly, during the early tumor stage, eosinophils tend to promote Th1-type response, but in advanced stages, they become more abundant and contribute to a Th2-skewed microenvironment, which supports tumor progression (43). In B-cell malignancies such as multiple myeloma, eosinophils exhibit pro-tumorigenic activity by promoting myeloma growth (44). While tissue eosinophilia is a well-recognized pathological hallmark of classical Hodgkin lymphoma, it is considered rare in NHL, possibly due to under-recognition. However, a recent study identified nodal marginal zone lymphoma (NMZL) as the subtype most frequently associated with tissue eosinophilia, followed by DLBCL (45). Our transcriptomic analysis revealed a significantly elevated eosinophil signal in ABC DLBCL, suggesting eosinophil infiltration as a potentially important feature contributing to molecular subclassification.

Tumor-infiltrating lymphocytes (TILs) play a key role in the TME in DLBCL (46), in which CD8^+^ are the main effector immune cells to deliver an anti-tumor response. In our study, high CD8^+^ T-cell abundance was significantly associated with improved OS, especially in ABC patients. Similar results were obtained using flow cytometry quantification (30). Although this is an important observation, the majority of CD8^+^ tumor-infiltrating lymphocytes in DLBCL have been reported to be terminally exhausted, characterized by high TIM-3 expression, an immunological state associated with poor response to CHOP-based chemotherapy (46).

In order to find some prognostic biomarkers from biological data with significance in a COO-dependent manner, we combined the Cox proportional hazards model and subsequent LASSO regression. We identified *CCL18* as an independent biomarker of adverse prognosis. CCL18 is known to be secreted by M2-polarized tumor-associated macrophages and is associated with immunosuppression and tumor progression in various cancers, including lymphomas (47, 48). In our validation cohort, *CCL18* expression was markedly enriched in M2-polarized macrophages compared with the M1 or M0 subtype. Given the well-established role of M2 macrophages in promoting an immunosuppressive tumor microenvironment, this finding suggests that *CCL18* may contribute to lymphoma progression by fostering pro-tumorigenic signaling and impaired antitumor immunity in ABC lymphomas.

Although increased CD8^+^ T-cell infiltration was associated with improved survival in our cohort, the concomitant overexpression of *CCL18* and epigenetic silencing of *MAPK10* indicate a complex interplay between tumor-intrinsic alterations and the immune microenvironment. CCL18, secreted by M2-polarized macrophages, is known to induce T-cell anergy and promote a Th2-skewed, immunosuppressive milieu that may limit the cytotoxic efficacy of infiltrating CD8^+^ lymphocytes (47, 48); *MAPK10* downregulation through promoter hypermethylation impairs apoptotic signaling and stress response pathways, contributing to immune evasion (29, 39, 40). Furthermore, our additional analyses indicate that the prognostic effect of CD8^+^ T-cell infiltration is critically modulated by MAPK10 status in a setting where CCL18 is expressed. In MAPK10^−^ tumors, the microenvironment appears to be driven by CCL18-associated immunosuppressive signaling, which diminishes or neutralizes the expected benefit of increased cytotoxic infiltration. However, when MAPK10 is expressed, this pattern reverses, and CD8-high tumors again show a survival advantage. This suggests that MAPK10 activity may counterbalance, at least in part, the immunomodulatory effects of CCL18, allowing the antitumor potential of cytotoxic T cells to re-emerge. Altogether, these findings highlight a complex immunoregulatory axis in DLBCL in which the interplay between CCL18, MAPK10, and CD8^+^ T cells determines whether the microenvironment is predominantly immunosuppressive or retains antitumor capacity.

These findings suggest that the simultaneous enrichment of CCL18-producing macrophages and the suppression of MAPK10 activity can create an immune-infiltrated yet functionally suppressed tumor microenvironment, explaining the paradoxical association between immune cell presence and poor clinical outcome observed in ABC DLBCL.

Finally, through connectivity mapping using the iLINCS platform, we identified proteasome inhibitors (MG-132 and bortezomib) as promising candidates for reversing the ABC transcriptomic signature. Bortezomib’s efficacy in ABC DLBCL has been reported in both preclinical models and clinical settings, particularly in combination regimens (49). Additionally, palbociclib, a selective CDK4/6 inhibitor, emerged as a candidate for targeting CDK4-overexpressing DLBC tumors in combination with a PI3K inhibitor, offering a rationally guided therapeutic opportunity based on our expression data (50). These findings build upon our previous work identifying PI3K and mTOR inhibitors as potential perturbagens for ABC lymphomas (29), further supporting the relevance of targeting these pathways. The top negatively correlated perturbagens (FDR < 0.01) formed three mechanistically defined clusters: proteasome inhibitors (MG-132 and 179324-69-7) with strong negative connectivity scores, HDAC inhibitors (trichostatin A and vorinostat), and MEK/CDK/TK pathway inhibitors (PD-0325901, palbociclib, cobimetinib, and dasatinib), suggesting robust potential for transcriptomic reversion of the ABC phenotype.

### 4.1 Study limitations and future directions

While our study provides valuable insights into the biology of ABC lymphomas and identifies potential prognostic biomarkers, several limitations should be acknowledged. First, the relatively modest sample size, particularly in the ABC subgroup (n = 20), limits statistical power for subgroup analyses. This limitation is exemplified by the borderline association observed for *MAPK10* gene expression in ABC cases (*p* = 0.10). Nevertheless, validation of our key findings in an independent cohort of 98 patients partially mitigates this limitation. Future studies involving larger, multicenter, ABC-enriched cohorts are essential to confirm the prognostic significance and clinical applicability of these biomarkers. Second, our data primarily demonstrate molecular correlations and do not yet establish a causal relationship. Functional studies, including gene knockdown or overexpression experiments, are necessary to determine whether *MAPK10* and *CCL18* act as bona fide therapeutic targets rather than solely prognostic indicators. Likewise, the iLINCS-identified compounds (proteasome and CDK4/6 inhibitors) need to undergo preclinical validation using patient-derived xenograft and organoid models to confirm efficacy and identify optimal combination strategies.

Despite these limitations, our findings provide a strong framework for future mechanistic and translational studies aimed at improving biomarker-guided therapies in ABC lymphomas.

In conclusion, our integrative transcriptomic and immunogenomic analyses define the molecular and immune landscape of ABC DLBCL and identify *MAPK10* and *CCL18* as potential prognostic biomarkers. Pyrosequencing confirmed *MAPK10* promoter hypermethylation and reduced gene expression, whereas high *CCL18* levels, predominantly produced by M2-polarized macrophages, were independently linked to inferior survival, particularly in ABC lymphomas. Although our findings are based on correlational molecular data and require functional validation, these findings provide clinically relevant insights into disease biology and establish a foundation for biomarker-guided precision treatment strategies in aggressive B-cell lymphomas.

## Data Availability

The datasets presented in this study can be found in online repositories. The names of the repository/repositories and accession number(s) can be found below: https://www.ncbi.nlm.nih.gov/, GSE306513.
