# Supplementary material for: CCL18: a potential immunosuppressive biomarker for prognosis in ABC diffuse large B-cell lymphoma
Source: Front Immunol. 2025 Dec 15;16:1693730. doi: 10.3389/fimmu.2025.1693730 (PMC12745396; doi:10.3389/fimmu.2025.1693730)
Supplement: Supplementary file 1 [file DataSheet1.docx]

**Supplementary Information**

**CCL18: A potential immunosuppressive biomarker for prognosis in ABC diffuse large B-cell lymphoma.**

**Authors:**

^1^Marta Rodríguez, ^2^Francisco Rojas-Vega, ^1^Jesus Frutos Díaz-Alejo, ^3^Ignacio Mahillo-Fernandez, ^4^Cristina Serrano, ^5^Alberto López, ^2^Teresa Morales-Ruiz, ^2^Teresa Roldan-Arjona, ^6^Joaquín Sánchez-García, ^7,8^Ana Río Machín, ^5^Daniel Morillo, ^5^M Angeles Pérez, ^5,7^Raúl Córdoba, ^5,7^Pilar Llamas-Sillero, ^1^Socorro María Rodríguez-Pinilla, ^7,9^Juana Serrano-López.

**Affiliations:**

^1^Pathology Department, Fundación Jiménez Díaz University Hospital, Madrid, Spain.

^2^Department of Genetics. University of Cordoba, Córdoba, Spain. Maimonides Biomedical Research Institute of Cordoba (IMIBIC), Córdoba, Spain. Reina Sofía University Hospital, Córdoba, Spain.

^3^ Bioestatistics and Epidemiology Unit. Instituto de Investigación Sanitaria-Fundación Jiménez Díaz University Hospital, Madrid, Spain

^4^Immunology department, Fundación Jiménez Díaz University Hospital, UAM, Madrid, Spain.

^5^Hematology department, Fundación Jiménez Díaz University Hospital, UAM, Madrid, Spain

^6^Hematology Department, Reina Sofía University Hospital/Maimonides Biomedical Research Institute of Córdoba (IMIBIC)/University of Córdoba, Spain.

^7^Experimental Hematology Lab, IIS-Fundación Jiménez Díaz, UAM, Madrid, Spain.

^8^Centre for Haemato-Oncology, Barts Cancer Institute, Queen Mary University of London, UK

^9^Facultad de Biomedicina, Universidad Alfonso X el Sabio (UAX), Villanueva de la Cañada, Spain.

**Correspondence :** [**juana.serrano@quironsalud.es**](mailto:juana.serrano@quironsalud.es)**;** [**juanselo@uax.es**](mailto:juanselo@uax.es)

Supplementary Table 1. Additional Genes added.

| **Reference Sequence** | **Gene Name** | **Reactome Annotations** |
| --- | --- | --- |
| NM_174908.3 | CCDC50 | Ubiquitin-dependent degradation of Cyclin D |
| NM_014207.2 | CD5 | TCR Signaling |
| NM_000573.3:4465 | CR1 | Complement cascade |
| NM_001006658.1:485 | CR2 | B Cell Receptor Activation |
| NM_194071.3 | CREB3L2 | Unfolded Protein Response |
| NM_001716.3:2618 | CXCR5 | Chemokine receptor binds chemokines |
| NM_016229.3 | CYB5R2 | Metabolism of steroids |
| NM_022552.4:2056 | DNMT3A | DNA Methylation |
| NM_002460.1 | IRF4 | Interferon Signaling |
| NM_002221.3 | ITPKB | Inositol phosphate metabolism |
| NM_014240.2 | LIMD1 | Regulation of TP53 Activity |
| NM_018717.4 | MAML3 | NOTCH signaling |
| NM_002417.2 | MKI67 | Cell Cycle Checkpoints |
| NM_000902.3 | MME | Neuropeptide degradation |
| XM_034274.14 | MYBL1 | Transcriptional regulation of white adipocyte differentiation |
| NM_002467.3 | MYC | MYC Active Pathway |
| NM_024865.2:1100 | NANOG | Transcriptional regulation of pluripotent stem cells |
| NM_006206.3:1925 | PDGFRA | Signaling by PDGF |
| NM_014143.3 | PDL1 | PD-1 signaling |
| NM_006875.3 | PIM2 | Signaling by Interleukins |
| NM_182907.1:310 | PRDM1 | Transcriptional regulation of cytokine genes |
| NM_001135664.1 | RAB7L1 | Vesicle-mediated transport |
| NM_004230.3 | S1PR2 | Sphingosine metabolism |
| NM_002997.4:2680 | SDC1 | Extracellular matrix organization |
| NM_001042518.1 | SERPINA9 | Regulation of Complement cascade |
| NM_003109.1:5970 | SP1 | Transcriptional regulation by SP1 |
| NM_022037.3:404 | TIA1 | mRNA Splicing |
| NM_012452.2 | TNFRSF13B | TNFR2 non-canonical NF-kB pathway |
| NM_152942.2 | TNFRSF8 | TNFR2 non-canonical NF-kB pathway |
| NM_000546.2 | TP53 | p53-Dependent DNA Damage Response; R-HSA-6804116: TP53 Regulates Metabolic Genes |

|  | **TOTAL DLBCL** | | **GCB** | | **ABC** | |
| --- | --- | --- | --- | --- | --- | --- |
| **Variable** | **HR (95% CI)** | **P** | **HR (95% CI)** | **P** | **HR (95% CI)** | **P** |
| ANGPT2 | 1.58 (1.02-2.46) | 0,042 | 0.97 (0.49-1.94) | 0,934 | 2.08 (1.13-3.85) | 0,019 |
| CCL18 | 2.09 (1.40-3.11) | <0.001 | 1.50 (0.87-2.59) | 0,144 | 7.89 (1.71-36.4) | 0,008 |
| CD47 | 1.96 (1.06-3.62) | 0,032 | 1.38 (0.70-2.73) | 0,348 | 2.72 (1.12-6.60) | 0,027 |
| EXO1 | 0.45 (0.23-0.87) | 0,018 | 0.01 (0.00-0.68) | 0,032 | 0.35 (0.11-1.08) | 0,067 |
| FOXP3 | 1.65 (1.02-2.68) | 0,042 | 1.98 (0.89-4.40) | 0,093 | 1.37 (0.77-2.41) | 0,283 |
| GPR160 | 1.50 (1.02-2.20) | 0,037 | 1.38 (0.79-2.40) | 0,262 | 1.50 (0.82-2.76) | 0,189 |
| HLADQA1 | 1.81 (1.12-2.93) | 0,015 | 1.90 (0.83-4.33) | 0,126 | 2.02 (0.92-4.41) | 0,079 |
| HLADQB1 | 1.51 (1.02-2.25) | 0,041 | 1.82 (0.81-4.08) | 0,149 | 3.24 (1.13-9.29) | 0,029 |
| IL18R1 | 1.66 (1.13-2.44) | 0,010 | 2.11 (0.88-5.02) | 0,093 | 1.48 (0.94-2.32) | 0,089 |
| IL2RA | 1.50 (1.08-2.08) | 0,015 | 1.53 (0.89-2.65) | 0,124 | 5.11 (1.43-18.3) | 0,012 |
| MAML2 | 1.69 (1.10-2.59) | 0,017 | 0.92 (0.45-1.88) | 0,823 | 2.19 (1.11-4.31) | 0,023 |
| MAPK10 | 0.23 (0.07-0.73) | 0,012 | 0.21 (0.04-1.07) | 0,061 | 0.29 (0.06-1.45) | 0,131 |
| MLH1 | 1.83 (1.07-3.13) | 0,026 | 1.12 (0.59-2.14) | 0,729 | 3.06 (1.16-8.08) | 0,024 |
| NLRC5 | 1.80 (1.06-3.07) | 0,030 | 2.29 (0.89-5.88) | 0,085 | 1.70 (0.86-3.36) | 0,129 |
| RELN | 1.64 (1.04-2.60) | 0,035 | 2.10 (0.85-5.21) | 0,110 | 1.60 (0.84-3.04) | 0,149 |
| TCF3 | 0.28 (0.12-0.68) | 0,005 | 0.41 (0.14-1.22) | 0,110 | 0.03 (0.00-0.72) | 0,030 |
| TLR1 | 2.00 (1.10-3.64) | 0,023 | 1.88 (0.68-5.20) | 0,223 | 2.59 (0.99-6.73) | 0,052 |
| TNKS | 1.43 (1.01-2.02) | 0,043 | 2.30 (1.17-4.51) | 0,015 | 1.39 (0.69-2.82) | 0,359 |
| TYMS | 1.58 (1.00-2.48) | 0,050 | 1.25 (0.64-2.44) | 0,508 | 1.69 (0.89-3.20) | 0,108 |
| ZAP70 | 2.15 (1.19-3.86) | 0,011 | 2.87 (1.11-7.40) | 0,029 | 1.65 (0.78-3.49) | 0,192 |

Supplementary Table 2. Univariate analysis for Overall Survival.

DLBCL: Diffuse Large B Cell Lymphoma. GCB: Germinal Center B Cell. ABC: Activated B Cell. HR: Hazard Ratio. CI: Confident Interval. P: p-value

Supplementary Table 3. Multivariante analysis for Overall Survival.

|  |  |  | 95% Conf. Int. | |  |
| --- | --- | --- | --- | --- | --- |
| Variable | Coefficient | HR | Lower | Upper | *p*-value |
| CCL18 | 0.626 | 1.871 | 1.251 | 2.799 | 0.002 |
| EXO1 | -0.745 | 0.475 | 0.227 | 0.993 | 0.048 |
| Likelihood ratio test = 15.87 on 2 df; p < 0.001 | | | | | |
| Wald test = 14.55 on 2 df; p < 0.001 | | | | | |
| Score (log-rank) test: 19.23 on 2df; p < 0.001 | | | | | |
| Log-Likelihood = -34.634 | | | | | |
| AIC = 73.3 | | | | | |
| C-Statistic = 0.81 (Leave-One-Out cross-validation) | | | | | |
| Proportional hazards test *p*-value: | | | | | |
| Global = 0.547 | | | | | |
| CC18 = 0.621 | | | | | |
| EXO1 = 0.349 | | | | | |

Supplementary Table 4. List of Perturbagenes.

| **PerturbagenId** | **Perturbagen** | **GeneTargets** | **Correlation** | **NoOfSignatures** | **pValue** | **zScore** |
| --- | --- | --- | --- | --- | --- | --- |
| BRD-K60230970 | MG-132 | PSMB1 | - | 2193 | 0 | 6.080.563.793 |
| BRD-K50691590 | 179324-69-7 | PSMB5, PSMB1, PSMB2, PSMB8, PSMB9, PSMB6, PSMA1, PSMA2, PSMA3, PSMA4, PSMA5, PSMA6, PSMA7, PSMA8, PSMB10, PSMB11, PSMB3, PSMB4, PSMB7, PSMD1, PSMD14, ADRM1, PSMC2, PSMC1, PSMC4, PSMC6, PSMC3, PSMC5, PSMD2, PSMD3, PSMD12, PSMD11, PSMD6, PSMD7, PSMD13, PSMD4, PSMD8, SEM1 | - | 2112 | 0 | 5.032.531.805 |
| BRD-A19037878 | Trichostatin A, Streptomyces Sp. | HDAC1, HDAC3, HDAC4, HDAC6, HDAC7, HDAC8, HDAC10 | - | 794 | 3,13E-192 | 3.024.394.039 |
| BRD-K81418486 | Vorinostat | HDAC1, HDAC2, HDAC3, HDAC6, HDAC8 | - | 1177 | 1,14E-172 | 2.871.779.982 |
| BRD-K49865102 | PD-0325901 | MAP2K1, MAP2K2 | - | 639 | 3,90E-129 | 2.499.029.058 |
| BRD-A75409952 | C23H24O8 | PIK3CA, PIK3CG, PLK1 | - | 785 | 6,57E-88 | 208.570.689 |
| BRD-K51313569 | Palbociclib | CDK4, CDK6, CCND1 | - | 198 | 3,77E-74 | 1.928.246.618 |
| BRD-A28746609 | BRD-A28746609 | TUBB, NR1I2 | + | 58 | 1,29E-73 | 1.921.871.607 |
| BRD-A05821830 | DOCETAXEL | NA | + | 42 | 1,42E-70 | 1.885.176.966 |
| BRD-K03390685 | Cobimetinib | MAP2K1, MAP2K2 | - | 35 | 2,96E-57 | 1.715.347.666 |
| BRD-K03601870 | Ixabepilone | TUBA4A, TUBB1, TUBB4A, TUBB8, TUBB, TUBA1A, TUBB4B, TUBB3, TUBA1B, TUBB2A, TUBA3E, TUBA1C, TUBB6, TUBB2B | + | 37 | 8,98E-53 | 1.654.306.291 |
| BRD-K49328571 | Dasatinib | ABL1, FYN, LCK, SRC, KIT, YES1, EPHA2, LYN, PDGFRB, BCR, HCK, FGR, FRK, BLK, SRMS | - | 662 | 4,23E-48 | 1.588.187.203 |
| BRD-K06858286 | Cabazitaxel | NA | + | 40 | 8,39E-47 | 1.569.343.646 |
| BRD-K29968218 | KX2-391 | SRC | + | 41 | 2,74E-45 | 1.547.053.649 |
| BRD-K24576554 | AT-9283 | JAK2, AURKA, AURKB, JAK3, ABL1, FLT3 | - | 86 | 6,44E-43 | 1.511.509.572 |
| BRD-K68164687 | Epothilone B (EPO906, Patupilone) | NA | + | 42 | 2,21E-41 | 1.488.023.129 |
| BRD-K68532323 | SB743921 | NA | + | 40 | 8,41E-41 | 1.479.072.907 |
| BRD-K74057757 | cortisone-acetate | TUBA4A, TUBB1, TUBB4A, TUBB8, TUBB, TUBA1A, TUBB4B, TUBB3, TUBA1B, TUBB2A, TUBA3E, TUBA1C, TUBB6, TUBB2B | + | 41 | 1,33E-39 | 1.460.354.603 |
| BRD-K43586850 | Lacidipine | NA | - | 38 | 7,03E-39 | 1.448.980.893 |
| BRD-K33379087 | Tivantinib | MET | + | 147 | 1,91E-38 | 14.420.963 |
| BRD-K61397605 | Romidepsin | NA | - | 38 | 5,99E-38 | 1.434.180.534 |
| BRD-K36970462 | Tonabersat | NA | + | 24 | 1,75E-36 | 1.410.575.823 |
| BRD-K41438959 | Perampanel | GRIA3, GRIA2, GRIA4, GRIA1 | - | 33 | 2,88E-36 | 1.407.066.841 |
| BRD-K77908580 | Entinostat | HDAC1, HDAC8, HDAC10, HDAC3, HDAC4, HDAC7, HDAC5, HDAC9, HDAC2, HDAC6, HDAC11 | - | 269 | 3,80E-36 | 1.405.095.531 |
| BRD-K44844162 | Taselisib | PIK3CD, PIK3R2, PIK3R1, PIK3CA, PIK3CB, PIK3CG, PIK3R5, PIK3R3 | - | 42 | 1,40E-33 | 1.362.614.486 |
| BRD-K64800655 | PHA-793887 | CDK1, CDK2, CDK4, CDK5, CDK7, CDK9, CDK14, CDK11B, CDK8, CDK3, CDK6, CDK16, CDK17, CDK18, CDK13, CDK10, CDK20, CDK15, CDK19, CDK12 | - | 224 | 1,88E-34 | 1.360.479.187 |
| BRD-K70511574 | HMN-214 | FLT3, KDR, KIT, FLT4, PDGFRB, CSF1R, FLT1, RET | + | 158 | 4,65E-33 | 1.353.828.783 |
| BRD-K09951645 | Dabrafenib | BRAF | - | 149 | 3,20E-31 | 1.322.381.928 |
| BRD-A66419424 | MLN9708 | NA | - | 38 | 8,71E-31 | 1.314.820.553 |

**Supplementary Figure 1**

**
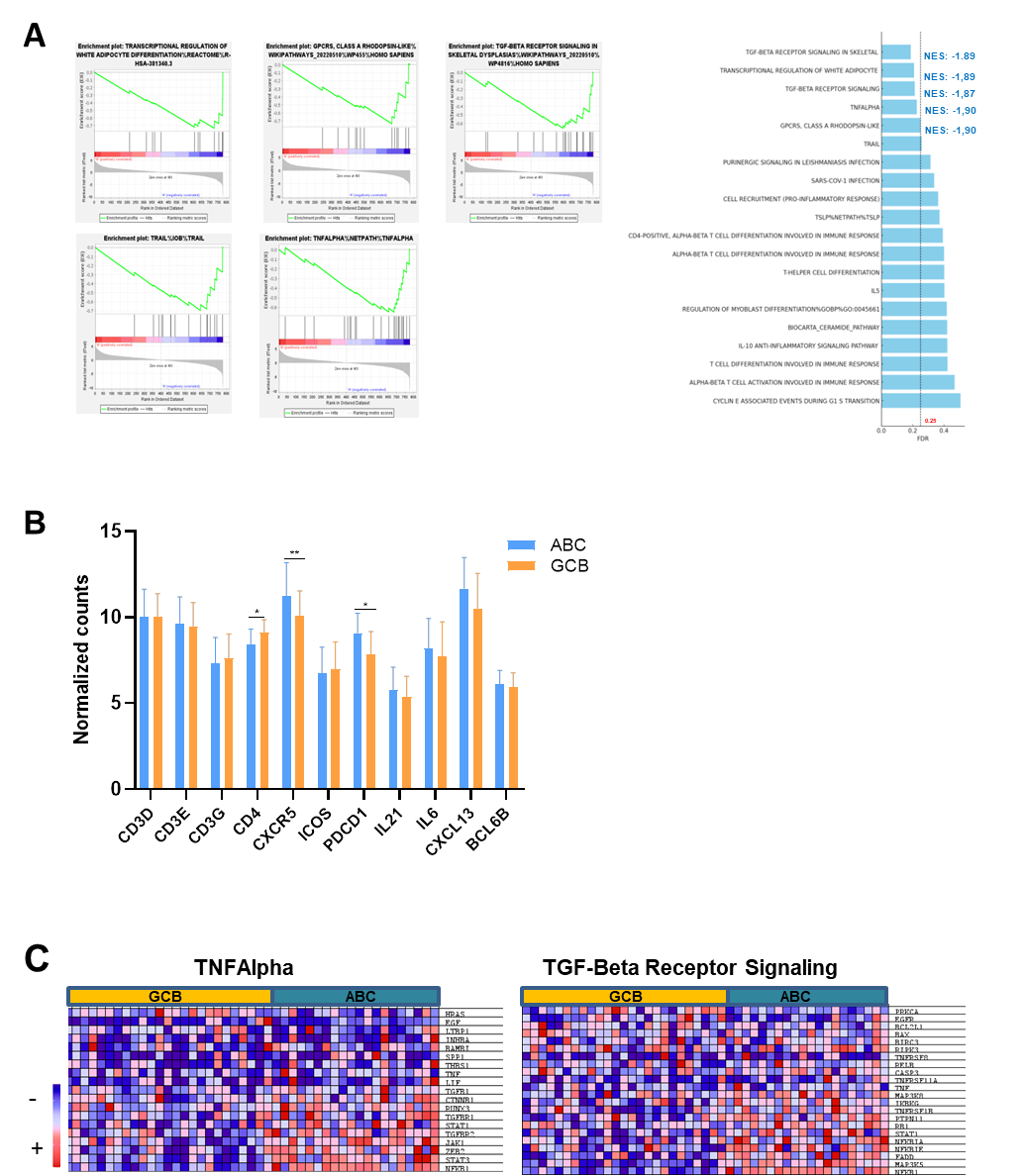
**

**Supplementary Figure 2**


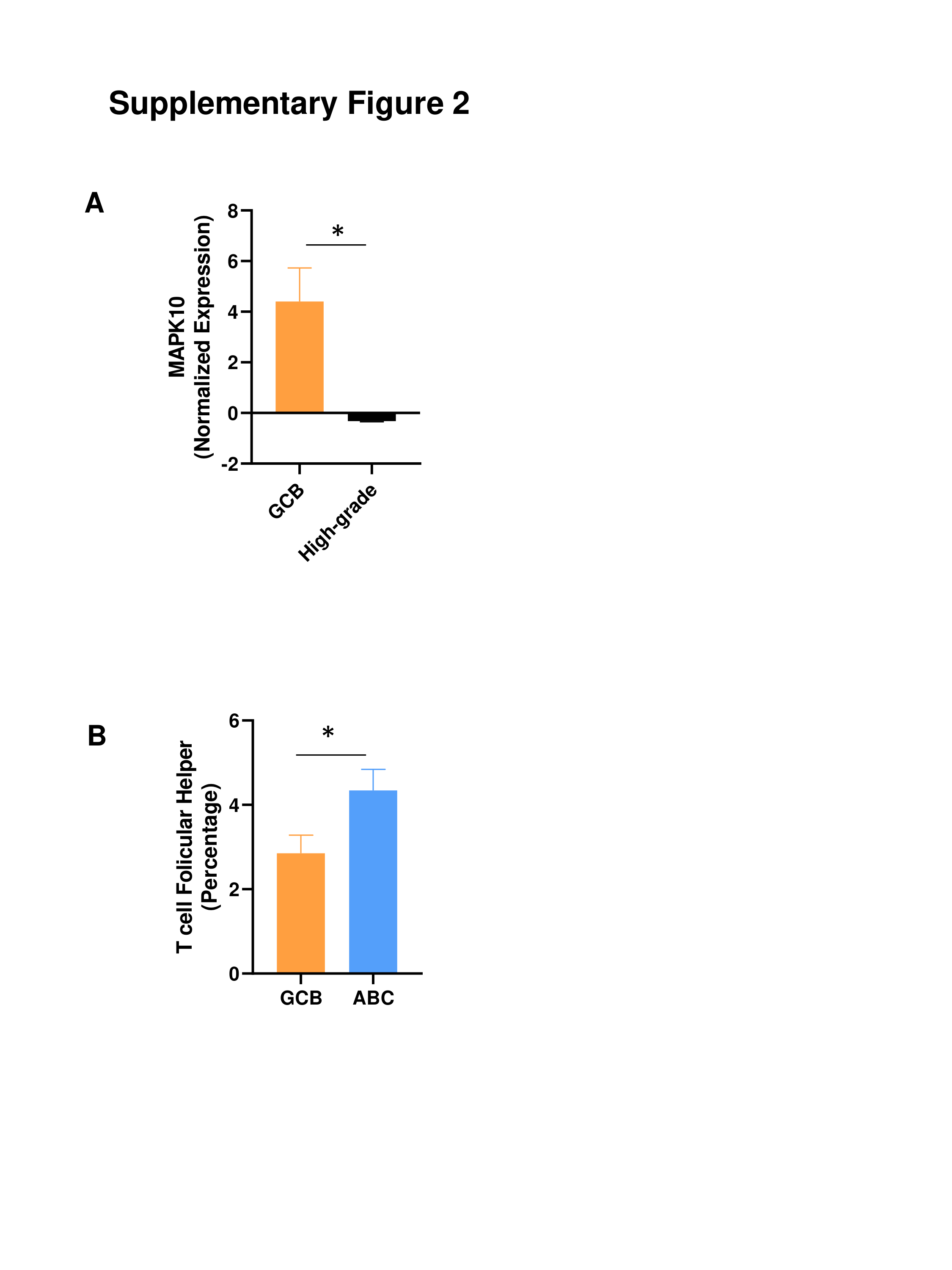


**Supplementary Figure 3**


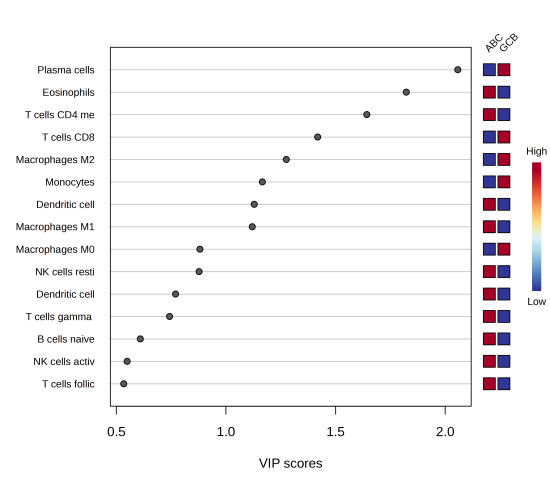

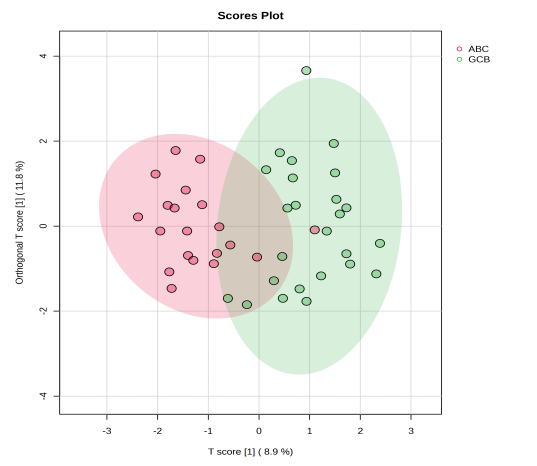


**A**

**B**

**C**

**

**Supplementary Figure 4**


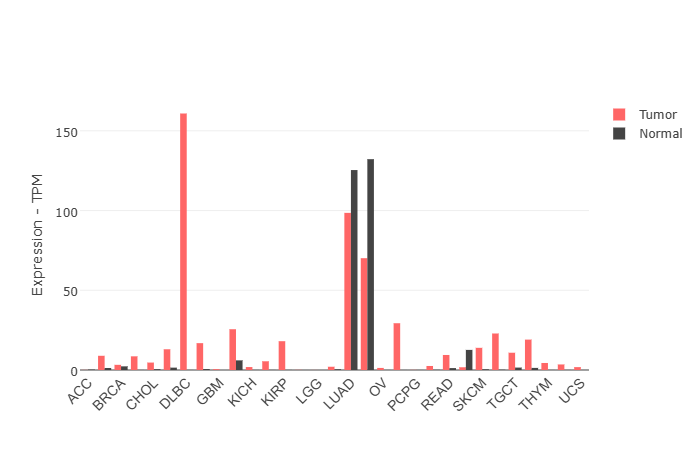


**GCB**

**P<0.69**

**GCB**

**P<0.58**

**P<0.12**

**P<0.19**

**C**

**D**

**E**

**F**

**CCL18**


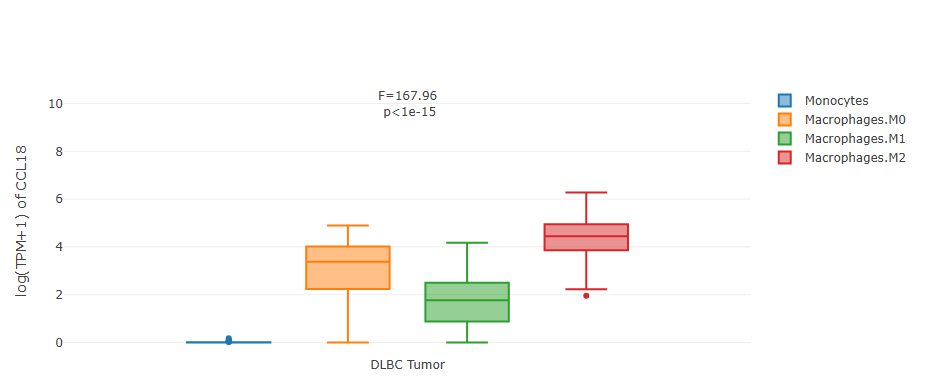

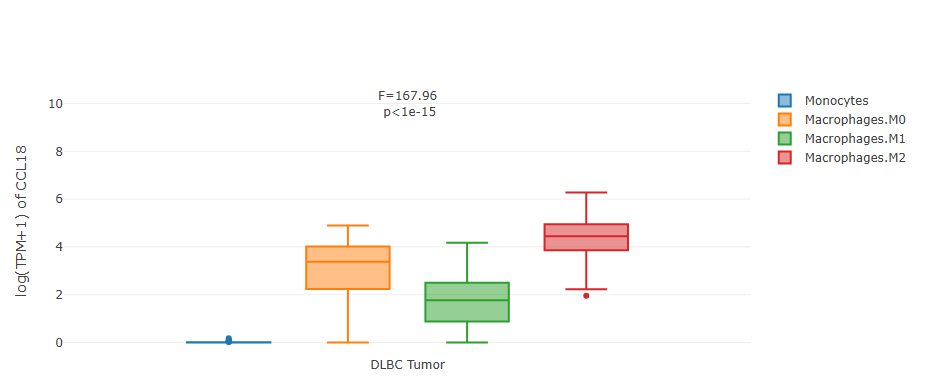


**F= 167.96**

**p<1^-15^**

**A**

**B**


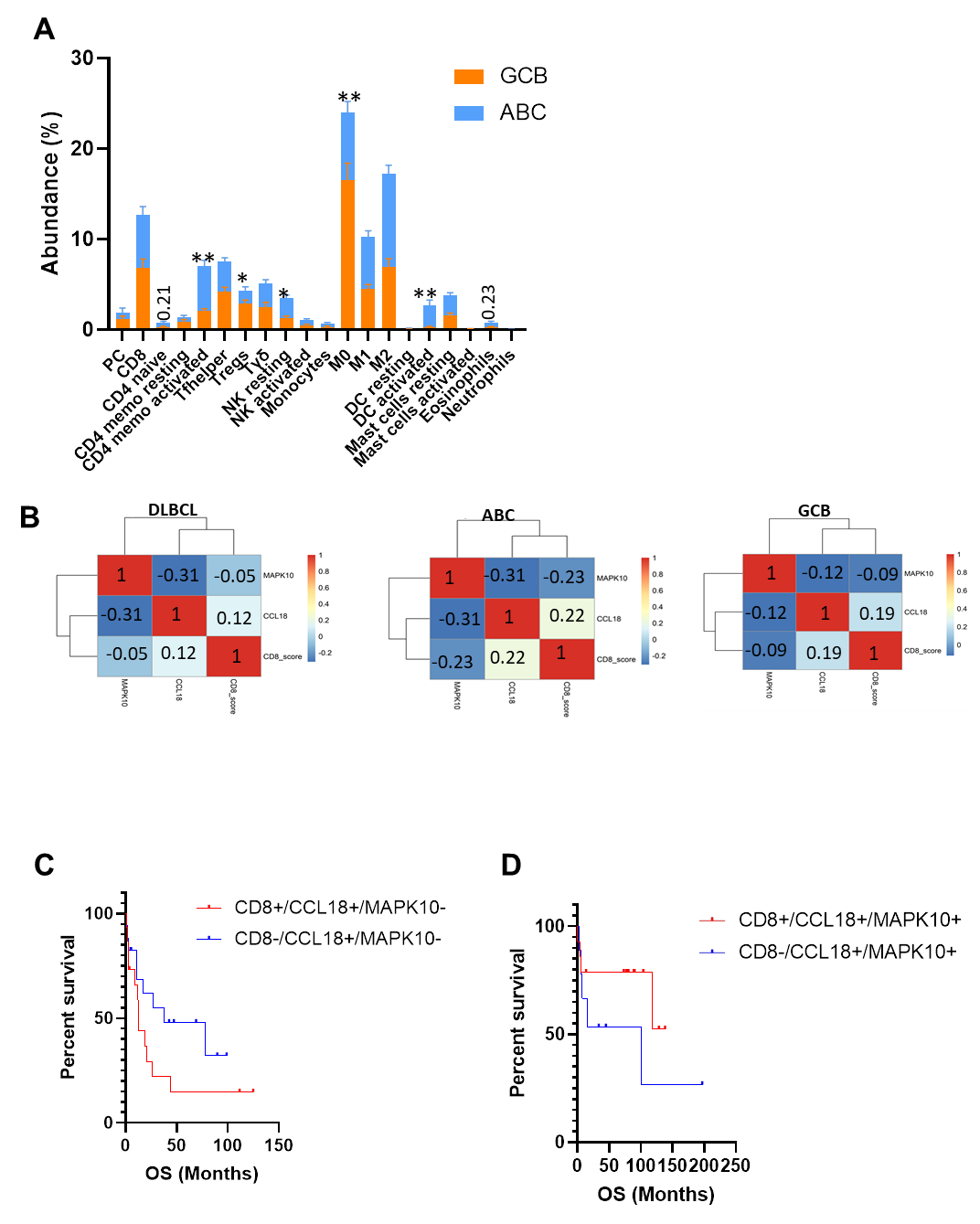
**Supplementary Figure 5**

**Supplementary Figure 6**


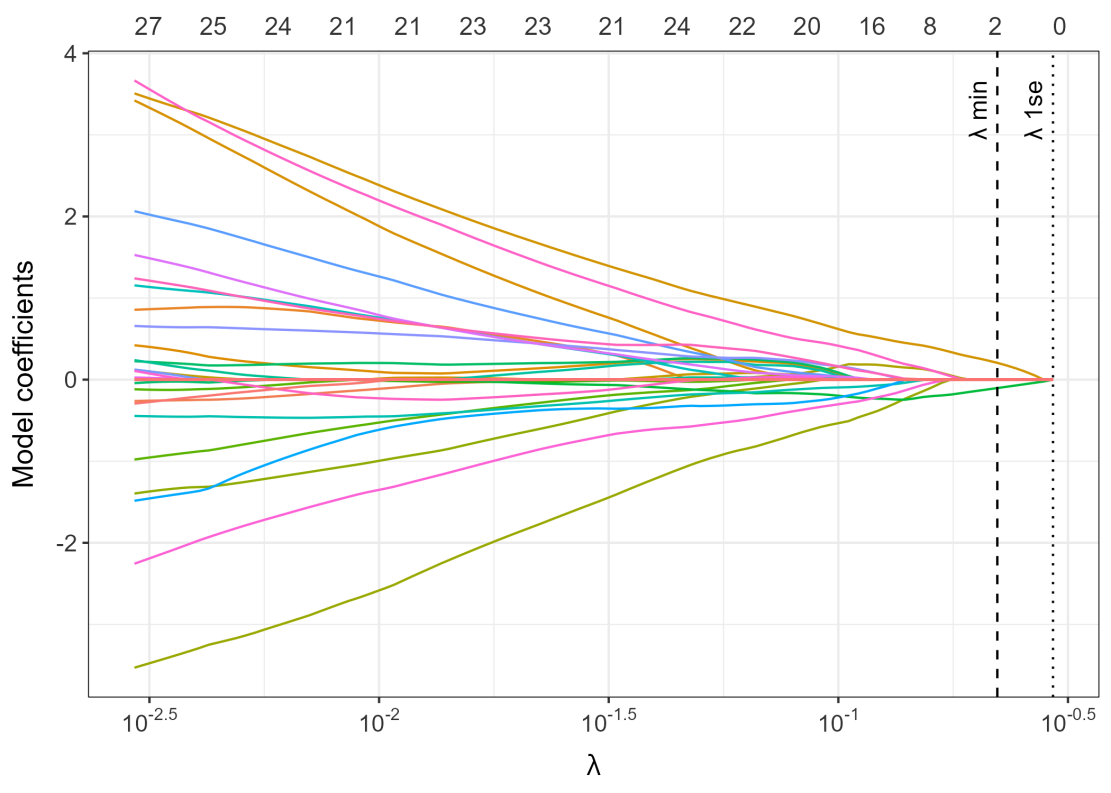

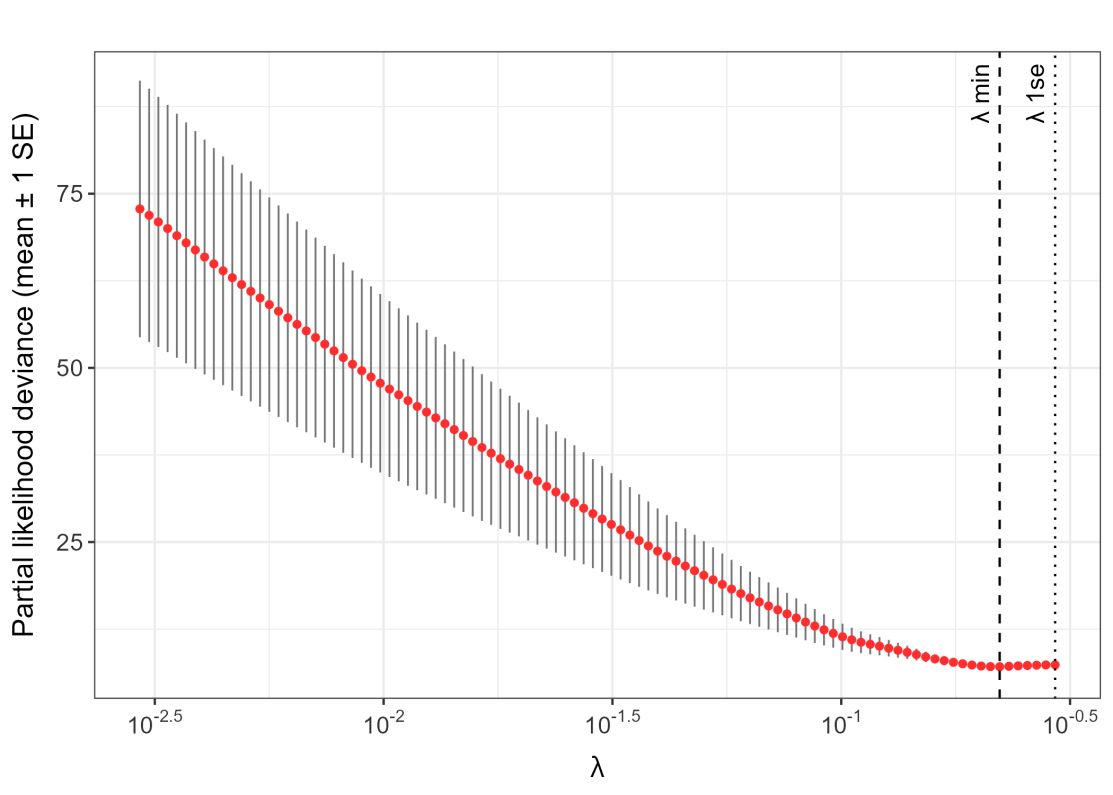


A

B

**Supplementary Figures Legends**

**Supplementary Figure 1**

**Enrichment and activity of immune-related pathways in ABC and GCB DLBCL subtypes.** (A) Gene Set Enrichment Analysis (GSEA) plots (left) and ranked enrichment scores (right) demonstrate the differential enrichment of immune signaling pathways between ABC and GCB DLBCL. Pathways such as TNFα signaling via NF-κB and TGF-β signaling are among the top enriched in ABC cases. (B) Bar plot showing normalized enrichment scores (NES) of hallmark immune-related gene sets in ABC (orange) and GCB (blue) subtypes. ABC subtype shows significantly higher enrichment in specific pathways including TNFα signaling and inflammatory responses (P < 0.05, Mann–Whitney test). (C) Heatmaps illustrating gene expression levels of key components in the TNFα signaling (left) and TGF-β receptor signaling (right) pathways across ABC and GCB samples. Expression intensity is color-coded (red = high, blue = low). Mann-Whitney test was used for group comparisons. (* p < 0.05, and ** *p* < 0.01 indicated by asterisk).

**Supplementary Figure 2**

**MAPK10 is downregulated in high-grade lymphoma compared to GCB-DLBCL.** (A) Bar graph showing normalized MAPK10 mRNA expression in GCB-type diffuse large B-cell lymphoma (GCB) versus high-grade lymphoma cases. (B) Significant increase in the percentage of T cells Folicular helper score in ABC. Data are presented as mean ± SEM, and Mann-Whitney test was used for group comparisons. (p < 0.05, indicated by asterisk).

**Supplementary Figure 3.**

**Transcriptomic discrimination between GCB and non-GCB DLBCL and enrichment of GCB-like features.** (A) Orthologal Partial Least Square (oPLS-DA) score plot showing clear separation between GCB (green) and ABC (pink) DLBCL subtypes based on gene expression profiles. Each point represents an individual sample, with distinct clustering patterns indicating subtype-specific gene expression signatures. (B) Variable Importance in Projection (VIP) scores ranking metabolites by their discriminatory power between GCB and ABC subtypes. Metabolites are ranked by VIP score magnitude, with higher scores indicating greater contribution to subtype discrimination. Color coding reflects the direction and strength of each gene expression's association with the respective DLBCL subtypes. (C) CD8⁺ T cell infiltration levels in GCB versus non-GCB DLBCL cases. Bar graph displaying CD8⁺ T cell percentages with individual data points overlaid. Non-GCB cases demonstrate significantly higher CD8⁺ T cell infiltration compared to GCB cases. Data presented as mean ± SEM. Statistical significance determined by unpaired t-test folowing Shapiro-Wilk normality testing (*p* <0.01)**.**

**Supplementary Figure 4.**

Distribution of CCL18 expression across DLBC tumor samples **and its relevance to GCB lymphomas. (A) CCL18 g**ene expression levels (TPM) across multiple TCGA cancer types comparing tumor and matched normal tissues. Bars represent median expression within each cohort, highlighting cancer-specific up- or down-regulation patterns, with notably elevated expression in DLBC (GEPIA2). (B) Boxplots showing CCL18 expression across distint macrophage-associated populations within DLBCL (GEPIA2). Among these subtypes, M2-type macrophages display the highest levels of CCL18 expression. (C-D) **Survival analysis of tumor microenvironment–related genes in the GCB subtype of DLBCL.** Kaplan–Meier curves represent overall survival (C-E) and progression-free survival (D-F) in patients classified as GCB (n = 49) within the validation cohort. Survival outcomes were stratified according to ***MAPK10*** (C-D) and ***CCL18*** (E-F) gene expression, comparing high (red) versus low (blue) expression groups. In contrast to the ABC subtype, neither *MAPK10* nor *CCL18* expression showed a significant impact on OS or PFS in the GCB subgroup, as indicated by log-rank *p*-values displayed in each plot.

**Supplementary Figure 5**

**(A)** Stack bars of relative abundance of immune cell subsets estimated by CIBERSORTx across ABC and GCB DLBCL samples. Bars represent mean proportions ± SEM for the 22 LM22-defined immune populations. **(B)** Correlation heatmaps showing the relationships among MAPK10, CCL18, and the CD8 score in the whole cohort (**left**), ABC subtype (**middle**), and GCB subtype (**right**). Color intensity reflects Spearman correlation coefficients. **(C–D)** Kaplan–Meier overall survival analyses stratified by CD8 infiltration (high vs. low), with further subdivision by MAPK10 status under conditions of CCL18 expression. In the **MAPK10⁻** subgroup (**C**), high CD8 infiltration did not confer a survival benefit. In contrast, in the **MAPK10⁺** subgroup (D), CD8-high cases showed improved survival relative to CD8-low cases. Not statistically significant differences were observed by log-rank test.

Supplementary Figure 6

LASSO Coefficient Regularization Path and Cross-Validation Error Curve. (A) Each colored line represents the trajectory of a single predictor’s coefficient as the regularization parameter (λ) increases from left to right (shown on a log₁₀ scale).The x-axis shows λ on a logarithmic scale; the y-axis shows the estimated coefficients. Vertical dashed and dotted lines mark λ_min and λ_1se, respectively.

(B) Cross-validated partial likelihood deviance versus λ for the LASSO-penalized Cox model. The x-axis shows λ on a logarithmic scale; points denote the mean deviance across folds and vertical bars indicate ±1 SE. Dashed and dotted vertical lines mark λ_min and λ_1se, respectively.
